# Simultaneous and Positively Correlated NET Formation and Autophagy in *Besnoitia besnoiti* Tachyzoite-Exposed Bovine Polymorphonuclear Neutrophils

**DOI:** 10.3389/fimmu.2019.01131

**Published:** 2019-05-22

**Authors:** Ershun Zhou, Iván Conejeros, Zahady D. Velásquez, Tamara Muñoz-Caro, Ulrich Gärtner, Carlos Hermosilla, Anja Taubert

**Affiliations:** ^1^Institute of Parasitology, Biomedical Research Center Seltersberg, Justus Liebig University Giessen, Giessen, Germany; ^2^Institute of Anatomy and Cell Biology, Justus Liebig University Giessen, Giessen, Germany

**Keywords:** *Besnoitia besnoiti*, PMN, NET formation, autophagy, cattle, AMPKα

## Abstract

Given that *B. besnoiti* tachyzoites infect host endothelial cells of vessels *in vivo*, they become potential targets for professional phagocytes [e.g., polymorphonuclear neutrophils (PMN)] when in search for adequate host cells or in case of host cell lysis. It was recently reported that *B. besnoiti*-tachyzoites can efficiently be trapped by neutrophil extracellular traps (NETs) released by bovine PMN. So far, the potential role of autophagy in parasite-triggered NET formation is unclear. Thus, we here analyzed autophagosome formation and activation of AMP-activated protein kinase α (AMPKα) in potentially NET-forming innate leukocytes being exposed to *B. besnoiti* tachyzoites. Blood was collected from healthy adult dairy cows, and bovine PMN were isolated via density gradient centrifugation. Scanning electron microscopy confirmed PMN to undergo NET formation upon contact with *B. besnoiti* tachyzoites. Nuclear area expansion (NAE) analysis and cell-free and anchored NETs quantification were performed in *B. besnoiti-induced* NET formation. Interestingly, tachyzoites of *B. besnoiti* additionally induced LC3B-related autophagosome formation in parallel to NET formation in bovine PMN. Notably, both rapamycin- and wortmannin-treatments failed to influence *B. besnoiti*-triggered NET formation and autophagosome formation. Also, isolated NETs fail to induce autophagy suggesting independence between both cellular processes. Finally, enhanced phosphorylation of AMP activated kinase α (AMPKα), a key regulator molecule of autophagy, was observed within the first minutes of interaction in tachyzoite-exposed PMN thereby emphasizing that *B. besnoiti*-triggered NET formation indeed occurs in parallel to autophagy.

## Introduction

*Besnoitia besnoiti* is a cyst-forming apicomplexan protozoan parasite that causes bovine besnoitiosis which is traditionally endemic in Africa and Asia. Recent continuous reports on bovine besnoitiosis outbreaks in several European countries (1–9) indicated a re-emergence and spread of this disease in Europe (10) and led to the classification as emerging disease by the European Food Safety Authority (EFSA) in 2010. Overall, bovine besnoitiosis has a detrimental impact on both, individual animal welfare (e.g., pain, oedemas, fever, abortion, orchitis, male infertility, severe skin lesions) and cattle industry (losses).

So far, very little data is available on early host innate immune reactions during primary acute *B. besnoiti* infections *in vivo* (11) and *in vitro* (12, 13) despite the fact that early host innate defense reactions should be critical for the outcome of the disease. In this sense, PMN play a pivotal role since they are the most abundant leukocyte population in blood and the first ones to be recruited to sites of infection. As reported for other mammalian species, bovine PMN own several efficient effector mechanisms to combat apicomplexan stages, such as phagocytosis (14), production of reactive oxygen species (ROS) (15) and *in vitro* excretion of antimicrobial peptides. Additionally, the release of neutrophil extracellular traps (NETs) in response to coccidian protozoa was reported (13, 16–18). NETs are commonly released via a PMN-derived cell death process known as NET formation (19). Suicidal NET formation was described as a NADPH oxidase (NOX)-dependent cellular mechanism which induces the extrusion of DNA and nuclear and cytoplasmic granule enzymes leading to the formation of DNA-rich networks being decorated with histones and various potent antimicrobial granular effector molecules, such as neutrophil elastase (NE), myeloperoxidase (MPO), lactoferrin, pentraxin, peptidoglycan recognition proteins, or calprotectin (19–22). A variety of invasive pathogens such as bacteria, virus, fungi, protozoan, and metazoan parasites, might either be immobilized within released sticky NET structures or be killed via local high concentration of antimicrobial histones, peptides, and proteases (16, 20, 23–25).

Classical suicidal NET formation is signaled via Raf-MEK-ERK-dependent pathways (18, 19, 26, 27). Besides NOX-dependent NET formation, NOX-independent NET formation also exists and seems to be linked to a substantial reduction of ERK1/2 activation and weak Akt activation, whilst p38 MAPK activation appears similar in both types of NET formation (28, 29). In addition to suicidal NET formation, PMN have also been shown to undergo vital NET formation without cell lysis, thus remaining viable and retaining the capability of active phagocytosis of bacteria (30). Furthermore, PMN seem able to release small-sized mitochondria-derived NETs without suffering cell death (31). So far, vital NET formation has not been described in response parasites. Suicidal NET formation was reported to be triggered by different protozoan parasites *in vitro* and *in vivo*, including *Plasmodium falciparum* (32), *Leishmania* spp., *Eimeria bovis* (16, 33)*, Eimeria arloingi* (17)*, Toxoplasma gondii* (34–36)*, Cryptosporidium parvum* (37), *Neospora caninum* (18, 38, 39), *Trypanosoma cruzi* (40), *Entamoeba histolytica* (41), and *B. besnoiti* (12, 13).

Autophagy is an essential intracellular degradation system, that recycles cell components as proteins and organelles and it is essential in the cellular response to stress (42). In neutrophils, autophagy has been described in PMN derived from mouse and human (43, 44). Interestingly, first evidences suggest that autophagy is necessary and can prime PMN to undergo NET formation (42, 45, 46). Besides other molecules, autophagy is regulated by the metabolic sensor molecule AMP activated kinase α (AMPKα) and by the mechanistic target of rapamycin (mTOR) (47). The processes of autophagy and NET formation appear to be linked in PMA-activated PMN and in sterile inflammation (44) by a mechanism which seems dependent on mTOR activation (48).

So far, *B. besnoiti*-mediated NET formation seems to be NOX-, NE- MPO-dependent and capable to efficiently hamper tachyzoites from active host cell invasion (13). On this regard, AMPK is been described as critical molecule of the autophagic process and governing critical functions in PMN as ROS production, chemotaxis and phagocytosis (49, 50). Despite this, nothing is known on the role of autophagy or autophagy-related molecules such as AMPKα in *B. besnoiti*-triggered NET formation.

Aim of the current study was to analyze the presence of autophagy during *B. besnoiti*-triggered suicidal NET formation. Therefore, we first confirmed NET formation induction by *B. besnoiti* tachyzoites and then showed that both, NET formation and autophagy are performed independent of rapamycin (stimulator of autophagy via mTOR binding), wortmannin (inhibitor of PI3K-mediated autophagy), treatments. In addition, we studied the release of extracellular DNA induced by *B. besnoitia* tachyzoites in presence of the autophagy-related molecules: LY294002 (inhibitor of PI3K-mediated autophagy) parthenolide (NF-κB inhibitor) or WP1130 (ubiquitinase inhibitor). Interestingly, NET formation and autophagosome formation occur simultaneously in tachyzoite-exposed PMN and is accompanied by a rapid phosphorylation of AMPKα.

## Materials and Methods

### Ethics Statement

This study was conducted in accordance to Justus Liebig University Giessen Animal Care Committee Guidelines. Protocols were approved by Ethic Commission for Experimental Animal Studies of Federal State of Hesse (Regierungspräsidium Giessen; A9/2012; JLU-No.521_AZ) and in accordance to European Animal Welfare Legislation: ART13TFEU and current applicable German Animal Protection Laws.

### Parasites

All NET formation -related experiments were performed with tachyzoite stage of the apicomplexan parasite *B. besnoiti* (strain Bb Evora04) which was initially isolated from the field in Portugal as previously reported (13).

### Host Cell Culture and *B. besnoiti* Tachyzoite Maintenance

Permanent Madin-Darby bovine kidney cells (MDBK) were used as host cells for *B. besnoiti* tachyzoite production *in vitro*. MDBK monolayers were cultured in 75 cm^2^ plastic tissue culture flasks (Greiner) at 37°C and 5% CO_2_ atmosphere until confluency using RPMI 1640 (Sigma) cell culture medium supplemented with 2% fetal bovine serum (FBS, Merck), 1% penicillin (500 U/ml) and streptomycin (500 mg/ml) (both Sigma-Aldrich). Confluent MDBK layers were infected with 2 × 10^6^ vital tachyzoites of *B. besnoiti*.

For experiments under physiological flow conditions, primary bovine umbilical vein endothelial cells (BUVEC) were isolated according to the method described by Taubert et al. (51). Briefly, umbilical cords retrieved from newborn calves via *Sectio caesarea* were enriched with 0.9% Hanks balanced salt solution (HBSS)-HEPES buffer (pH 7.4; Gibco) supplemented with 1% penicillin (500 U/ml; Sigma-Aldrich) and streptomycin (500 mg/ml; Sigma-Aldrich) and kept at 4°C until use. For isolation of host endothelial cells, the lumen of umbilical veins were infused with 0.025% collagenase type II solution (Worthington Biochemical Corporation). Veins were ligated and incubated for 20 min at 37°C and 5% CO_2_ atmosphere. Then, veins were gently massaged and collagenase-cell suspensions were harvested in 50-ml plastic tubes (Nunc) containing 1 ml FCS (Gibco) to inactivate collagenase type II. After two centrifugations (400 × *g*, 10 min, 4°C), endothelial cells were resuspended in complete ECGM (endothelial cell growth medium; PromoCell), plated in 25 or 75 cm^2^ plastic culture flasks (Greiner) and cultured at 37°C and 5% CO_2_ atmosphere until confluency. For flow assays, BUVEC were grown on Thermanox® coverslips (Nunc) until confluency.

### Isolation of Bovine PMN

Healthy adult dairy cows (*n* = 9) served as blood donors. Animals were bled by puncture of jugular vein and 30 ml blood was collected in 12 ml heparinized sterile plastic tubes (Kabe Labortechnik). Approximately 20 ml of heparinized blood were diluted in 20 ml sterile PBS with 0.02% EDTA (Sigma-Aldrich), layered on top of 12 ml Biocoll® separating solution (density = 1.077 g/l; Biochrom AG) and centrifuged (800 × *g*, 45 min). After removal of plasma and mononuclear cells, the cell pellet was suspended in 25 ml bi-distilled water and gently mixed during 40 s to lyse erythrocytes. Osmolarity was rapidly restored by adding 4 ml of 10 × Hanks balanced salt solution (Biochrom AG). For complete erythrocyte lysis, this step was repeated twice and PMN were later suspended in sterile RPMI 1640 medium (Sigma-Aldrich). PMN counts were analyzed in a Neubauer haemocytometer. Finally, freshly isolated bovine PMN were allowed to rest at 37°C and 5% CO_2_ atmosphere for 30 min until further use.

### Scanning Electron Microscopy (SEM)

Bovine PMN were co-cultured with vital *B. besnoiti* tachyzoites (ratio 1:4) for 60 min on coverslips (10 mm diameter; Thermo Fisher Scientific) pre-coated with 0.01% poly-_L_-lysine (Sigma-Aldrich) at 37°C and 5% CO_2_. After incubation, cells were fixed in 2.5% glutaraldehyde (Merck), post-fixed in 1% osmium tetroxide (Merck), washed in distilled water, dehydrated, critical point dried by CO_2_-treatment and sputtered with gold. Finally, all samples were visualized via a Philips® XL30 scanning electron microscope at the Institute of Anatomy and Cell Biology, Justus Liebig University Giessen, Germany.

### Immunofluorescence Microscopy Analyses for Visualization of *B. besnoiti*-Triggered NETosis

Bovine PMN were co-cultured with *B. besnoiti* tachyzoites (ratio 1:4) for 3 h (37°C and 5% CO_2_ atmosphere) on 0.01% poly-_L_-lysine pretreated coverslips (15 mm diameter, Thermo Fisher Scientific), fixed by adding 4% paraformaldehyde (Merck) and stored at 4°C until further experiments.

For NET visualizing, Sytox Orange (Life Technologies) was used to stain DNA and anti-histone (clone H11-4, 1:1,000; Merck Millipore #MAB3422), anti-NE (AB68672, 1:1,000, Abcam), or anti-MPO (orb11073, 1:1,000, Byorbit) antibodies were used to stain specific proteins on ETs structures. Therefore, fixed samples were washed three times with PBS, blocked with 1% bovine serum albumin (BSA, Sigma-Aldrich) for 30 min at RT and incubated with corresponding primary antibody solutions for 1 h at RT. Thereafter, samples were washed thrice with PBS and incubated in secondary antibody solutions (Alexa Fluor 488 goat anti-mouse IgG or Alexa Fluor 488 goat anti-rabbit IgG, both Life Technologies, 60 min, 1:1,000, RT). Finally, samples were washed thrice in PBS and mounted in anti-fading buffer (ProLong Gold Antifade Mountant; Thermo Fisher Scientific). Visualization was achieved using an inverted IX81 fluorescence microscope equipped with an XM 10 digital camera (Olympus).

### Extracellular DNA-Based Quantification of NETs

Bovine PMN were suspended in medium RPMI 1640 lacking phenol red and serum, confronted with vital *B. besnoiti* tachyzoites (96-well plates, duplicates) at a final PMN:tachyzoites ratio of 1:4 (2 × 10^5^ PMN + 8 × 10^5^ *B. besnoiti* tachyzoites). Samples were incubated at 37°C and 5% CO_2_. For the autophagy-related experiments, bovine PMN were pretreated with different concentrations of rapamycin (10, 50, 100, 200 nM), wortmannin (10, 50, 100, 200 nM), LY294002 100 μM, parthenolide 90 μM, or WP1130 5 μM for 30 min, then stimulated by *B. besnoiti* tachyzoites at a 1:4 PMN:tachyzoites ratio for 3 h. After incubation, samples were treated with 0.5 U/ml micrococcal nuclease (New England Biolabs) for 15 min and pelleted (300 × *g*, 5 min). Supernatants were collected for NET quantification which was performed by Picogreen®-based fluorometric measurements (19).

NETs are divided into two distinct forms: one is released away from neutrophils named cell-free NETs, and the other is those that are anchored to neutrophils (namely anchored NETs). For “cell free”- and “anchored”-NETs determination according to Tanaka et al. (52), the plate was directly centrifuged at 300 × *g* for 5 min after incubation. The supernatants were transferred into a new 96-well plate to measure “cell-free”-NETs and the pellets were used for “anchored”-NETs estimation. For both sampling methods, a 1:200 dilution of Pico Green® (Invitrogen) in 10 mM Tris base buffered with 1 mM EDTA was added to each well (50 μl), and then extracellular DNA was detected and quantified by PicoGreen®-derived fluorescence intensities using an automated multiplate reader (Varioskan, Thermo Scientific) at 484 nm excitation/520 nm emission.

### Estimation of “Anchored” NETs on *B. besnoiti*-Infected BUVEC Under Physiological Flow Conditions

BUVEC (*n* = 3) were cultured on Thermanox® (Nunc) coverslips pre-coated with bovine fibronectin (10 μg/ml, 2 h RT; Sigma-Aldrich) until confluency and infected with 2.5 × 10^5^ freshly isolated *B. besnoiti* tachyzoites. Twenty-four h. p. i., coverslips were washed to remove residual tachyzoites and placed into a parallel flow chamber (53). Bovine PMN (2.5 × 10^6^ PMN in 500 μl medium) were perfused into the system at a constant wall shear stress of 1.0 dyn/cm^2^ (syringe pump sp100i® World Precision Instruments). For “anchored”-NET formation visualization, coverslips were fixed, washed, and stained for DNA and histones as described above. Images were taken under an inverted fluorescence microscope (DM IRB; Leica) equipped with a digital camera (Olympus).

### Nuclear Decondensation-Based Quantification of NETs

Nuclear expansion-based quantification of NETs was performed according to the method described by Papayannopoulos et al. (54). Briefly, bovine PMN (*n* = 3) were pretreated with rapamycin (50 nM), wortmannin (50 nM) or plain medium (RPMI 1640, Sigma-Aldrich) for 30 min and then exposed to *B. besnoiti* tachyzoites for 3 h at a 1:4 PMN:tachyzoites ratio. After incubation, PMN were fixed by 4% paraformaldehyde (Merck) and stained with 5 μM Sytox Orange® (Life Technologies) for 30 min at RT. Five images were captured randomly for each condition using an inverted fluorescence microscope (Olympus IX 81) and nuclear area size of single cells was analyzed using ImageJ® software as described by Gonzalez et al. (55). Cells that presented decondensed nucleus and exceeded the threshold of 50 μm^2^ were considered as PMN undergoing NET formation. Overall, 1,200–1,700 PMN were analyzed for each experimental condition in samples from three different donors.

### Autophagosome Detection by Immunofluorescence Analysis

LC3 protein is a marker for autophagosomes (56) with LC3-I being cytosolic and LC3-II being membrane-bound and enriched in the autophagic vacuole. Analysis of autophagosome formation in PMN was performed according to Itakura and McCarty (48). In brief, bovine PMN (*n* = 3) were deposited on poly-_L_-lysine (0.01%) pre-treated coverslips (15 mm diameter, Thermo-Fisher scientific), pretreated with rapamycin (50 nM) or wortmannin (50 nM) for 30 min before being exposed to *B. besnoiti* tachyzoites at a 1:4 PMN:tachyzoite ratio for 3 h. After incubation, cells were fixed with 4% paraformaldehyde (10 min), permeabilized by ice cold methanol treatment (3 min at 4°C) and blocked with blocking buffer (5% BSA, 0.1% Triton X-100 in sterile PBS; all Sigma-Aldrich) for 60 min at RT. Thereafter, cells were incubated overnight at 4°C in anti-LC3B antibody solution (cat#2775 Cell Signaling Technology) diluted 1:200 in blocking buffer. After incubation, samples were washed thrice with PBS and incubated 30 min in the dark and RT in a 1:500 dilution of goat anti-rabbit IgG conjugated with Alexa Fluor 488 (Invitrogen). After three washes in PBS, samples were mounted in Prolong Anti-fading reagent with DAPI® (Invitrogen) on glass slides and images were taken applying confocal microscopy (Zeiss LSM 710). To estimate LC3B-positive cells, the background fluorescence signal was determined in control conditions for FITC (green) and DAPI (blue) channels. Image processing was carried out with Fiji ImageJ^®^ using Z-project and merged channel plugins and restricted to overall adjustment of brightness and contrast.

### Immunoblotting-Based Analysis of LC3B- and AMPK-Expression in Bovine PMN

Proteins from tachyzoite-exposed and non-exposed bovine PMN were extracted by lysing 5 × 10^6^ PMN using a ultrasound sonicator (20 s, 5 times) in RIPA buffer (50 mM Tris-HCl, pH 7.4; 1% NP-40; 0.5% Na-deoxycholate; 0.1% SDS; 150 mM NaCl; 2 mM EDTA; 50 mM NaF, all Roth) supplemented with a protease inhibitor cocktail (Sigma-Aldrich). The samples were centrifuged (10,000 × g, 10 min, 4°C) to sediment intact cells and nuclei, the supernatant was transferred to new tubes and the protein content was quantified via Coomassie Plus Assay Kit (Thermo Scientific) according to the manufacturer's instructions. For immunoblotting, samples were supplemented with 6 M urea. After boiling (95°C, 5 min), 60 μg of total protein/slot were electrophoresed in 12 or 15% polyacrylamide gels (100 V, 90 min) using a Mini-PROTEAN Tetra Cell system (Biorad). Proteins were then transferred (300 mA, 2h) to polyvinylidene difluoride (PVDF) membranes (Millipore) using a semidry blotting instrument (Mini-transfer blot, Biorad). Samples were first incubated in blocking solution (3% BSA in TBS containing 0.1% Tween, all Sigma-Aldrich) (1 h, RT) and then overnight at 4°C in anti-LC3B (Cat#2775, 1:1,000, Cell Signaling), anti-Atg5 (Cat#ab108327, 1:1,000 abcam), and anti-AMPKα T172 (Cat#5832, 1:1,000 Cell Signaling) antibody solution diluted in blocking solution. Detection of vinculin (Cat#sc-73614, 1:1,000, Santa Cruz) was used for the normalization of the sample. Signal detection was accomplished by applying solutions of corresponding secondary antibodies conjugated with peroxidase (Cat#31430, 1:40,000 and Cat#31460, 1:40,000; both Pierce) and enhanced chemiluminescence detection system (ECL® plus kit, GE Healthcare). Protein signals were recorded in a ChemoCam Imager® (Intas Science Imaging). Protein sizes were controlled by a protein ladder (PageRuler® plus prestained protein ladder ~10–250 kDa; Thermo Fisher Scientific). Quantification of protein band intensities was performed by the use of Image J® software (Fiji version using gel analyzer plugin).

### Statistical Analysis

Statistical significance was defined by a *p* value < 0.05. *p* value were determined by applying non-parametric analyses: Mann-Whitney test when two experimental conditions were compared and Kruskal-Wallis test followed by Dunn's *post-hoc* test for multiple comparisons. Correlation between LC3B-positive and NETotic PMN was determined by Spearman correlation test. All graphs (mean ± SD) and statistical analyses were generated by the use of Graph Pad software (v. 7.03).

## Results

### Visualization of *B. besnoiti*-Triggered NETs in Bovine PMN

**Figure 1:**
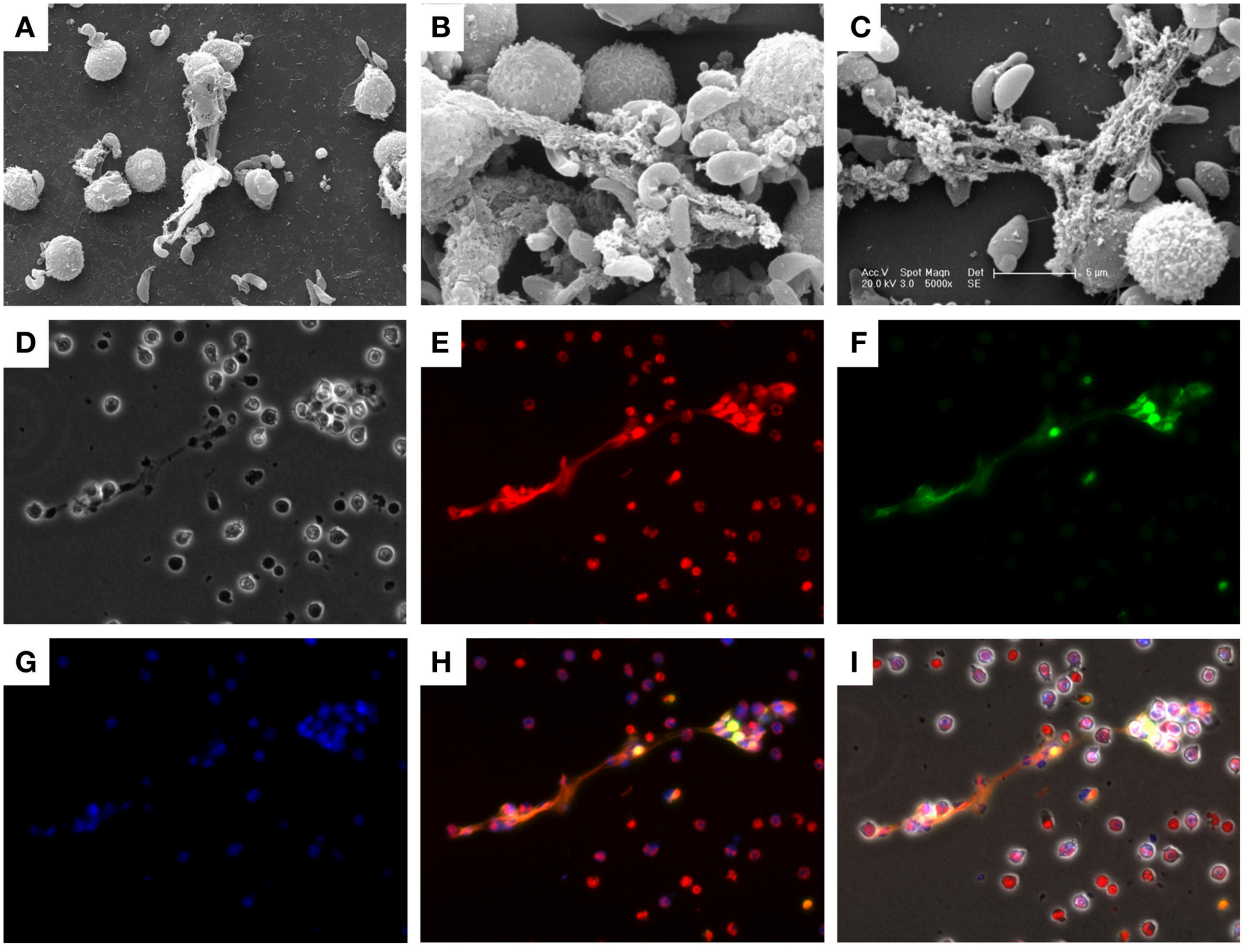
*Besnoitia besnoiti* tachyzoite-induced NET formation in bovine PMN. Co-cultures of bovine PMN and *B. besnoiti* tachyzoites were fixed and analyzed by scanning electron microscopy (SEM) analysis. **(A–C)** NETs, defined as chromatin extracellular structures forming a meshwork in contact with the tachyzoites, were confirmed and visualized via immunostaining. **(D)** Phase contrast image; **(E)** DNA staining; Sytox Orange; **(F)** histone (H11-4) staining; **(G)** neutrophil elastase (NE) staining; **(H)** Merged image of **(E–G)**; **(I)** Merged image of all channels.

SEM analysis showed that bovine PMN (Figure 1) exposed to vital *B. besnoiti* tachyzoites released NET-like structures and many *B. besnoiti* tachyzoites were firmly trapped by these filaments (Figures 1A–C). To verify that bovine PMN were indeed undergoing NET formation, the main components of NETs [i.e., histones (H11-4) and NE] were visualized by immunostaining. Co-localization analyses of extracellular DNA being adorned with H11-4, NE (Figures 1D–I) in parasite-entrapping structures confirmed classical characteristics of NETs. Furthermore, tachyzoites were entangled in these NETs structures confirming the observations in SEM analysis (Figures 1D,I; Control condition is shown in Figures S6 and S7).

**Figure 2:**
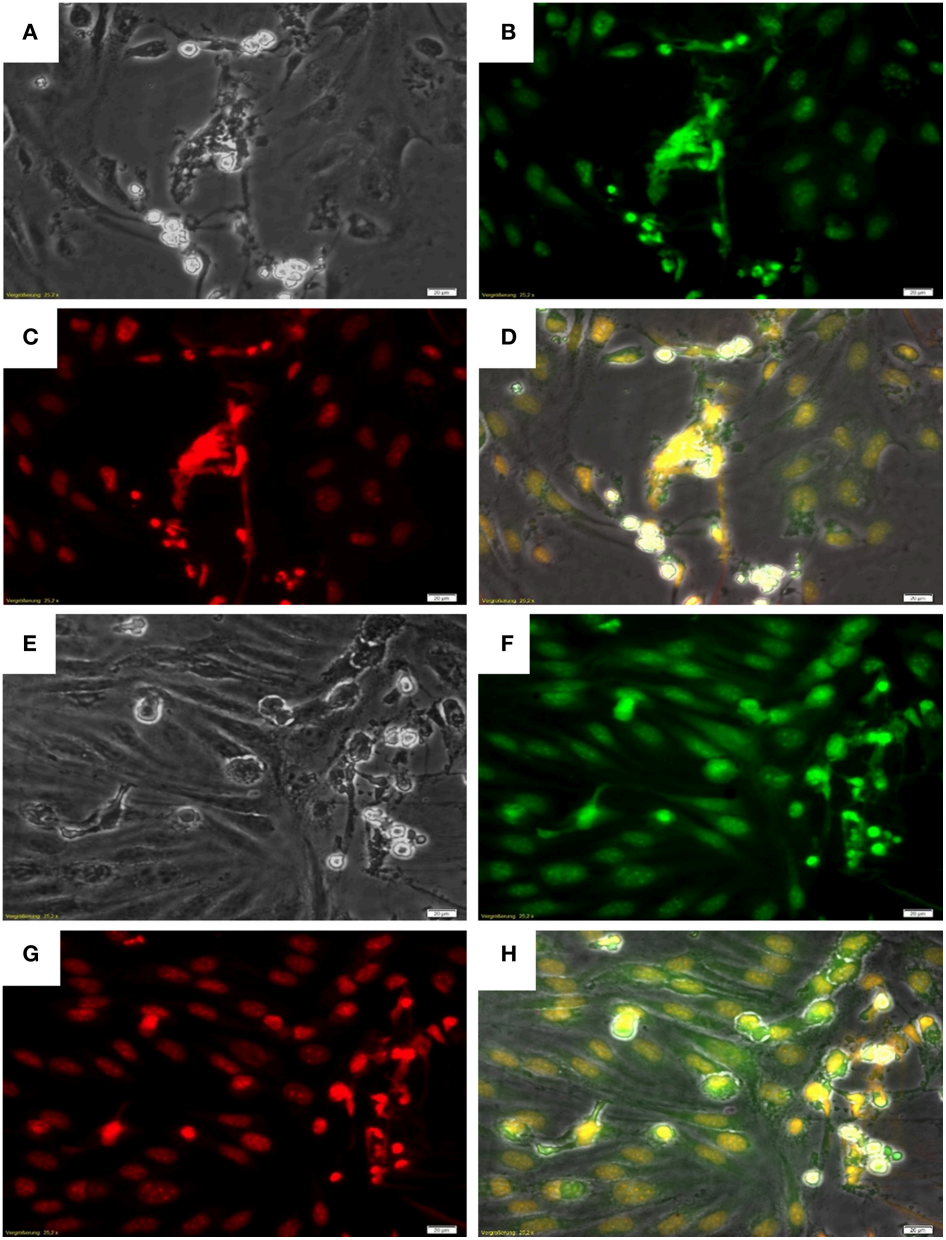
NET formation by PMN on *Besnoitia besnoiti*-infected BUVEC under physiological flow conditions. *B. besnoiti*-infected BUVEC on Thermanox coverslips were placed into the parallel plate flow chamber and bovine PMN were perfused into the system at a constant wall shear stress of 1.0 dyn/cm^2^. For NET visualization, the coverslips were fixed and stained with Sytox Orange jointly with an anti-histone antibody. **(A,E)** Phase contrast images; **(B,F)** histone staining with anti-histone antibody; **(C,G)** DNA staining using Sytox Orange; **(D,H)** Merged images: DNA (red), histones (green) and phase contrast.

Given that *B. besnoitia* tachyzoites develop within endothelial host cells, we wondered whether infected endothelium would also contribute to NET formation. Therefore, we chose an experimental approach which mimicked the *in vivo* situation in a small vessel: controlled physiological flow condition of 1.0 dyn/cm^2^ shear stress was applied on endothelial cell layers and a fixed number of PMN were floating over *B. besnoiti*-infected endothelial cells in parallel plate chamber assay (53). Under these flow conditions, bovine PMN also underwent “anchored”-NET formation on *B. besnoiti*-infected BUVEC which was also corroborated by co-localization of extracellular DNA decorated with histones (Figure 2).

### Effects of Autophagy on *B. besnoiti*-Stimulated NET Formation in Bovine PMN

To investigate effects of autophagy on *B. besnoiti*-triggered bovine NET formation, initially we used the mTOR-mediated autophagy inducer rapamycin (48) and the PIK3-mediated autophagy inhibitor wortmannin (57).

**Figure 3:**
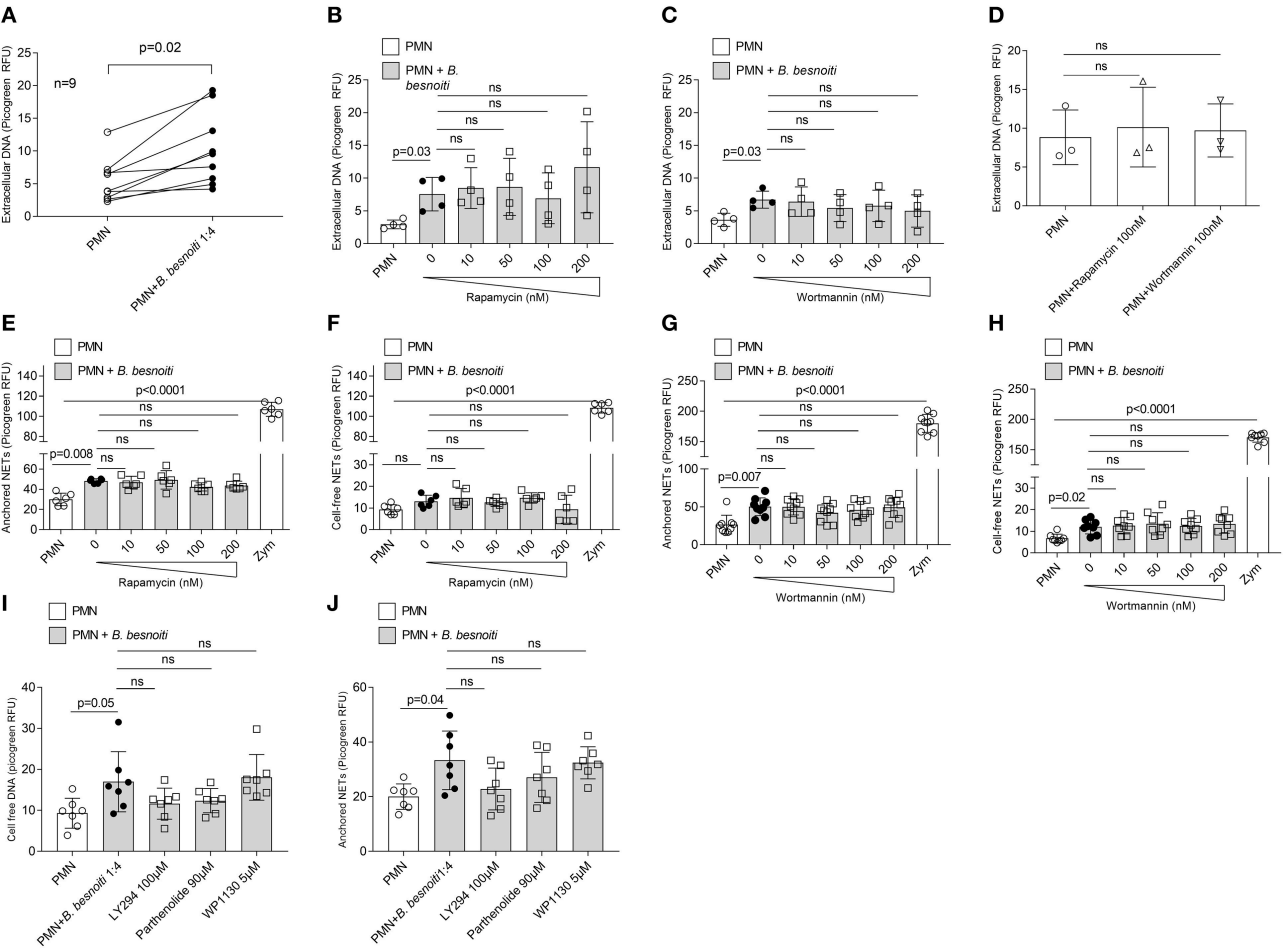
Effects of rapamycin and wortmannin treatments on *Besnoitia besnoitia*-triggered NET formation as detected by PicoGreen-based quantification. Bovine PMN were pre-treated with increasing concentrations of rapamycin and wortmannin for 30 min and then exposed to *B. besnoiti* tachyzoites for 3 h. After incubation, the samples were analyzed as follows. **(A–D)** samples were treated with micrococcal nuclease for 15 min, thereafter centrifuged and extracellular DNA present in the supernatants were analyzed using PicoGreen-based quantification. **(E–H)** samples were centrifuged immediately and the supernatants were analyzed for “cell free” NETs. Moreover, remaining pellets located at the bottom were investigated for “anchored” NETs. For both approaches, extracellular DNA was detected and quantified via PicoGreen-derived fluorescence intensities. In all assays, zymosan treatments (1 mg/ml) were used for positive controls. Statistical analyses were performed by Kruskal-Wallis test followed by Dunn's test for multiple comparisons. *p*-values are indicated in the graphs (Mean ± SD).

In a first experimental series, NET formation was measured based on PicoGreen®-derived fluorescence intensities as previously described (18, 19) thereby rather targeting late phase of NETosis. Overall, confrontation of bovine PMN with *B. besnoiti* tachyzoites resulted in a significant increase of NET formation when compared to control groups (*p* = 0.02–0.03; Figures 3A–C). Given that we always experience high individual variations in NET-related assays, the reactions induced in PMN derived from each animal (*n* = 9) are also depicted (Figure 3A). However, parasite-mediated NET formation was neither affected by rapamycin (tested in a range from 10 to 200 nM, Figure 3B) nor by wortmannin treatments (tested in the same range of concentration, Figure 3C). This lack of effect was also observed when non-stimulated PMN were treated with these compounds (100 nM) for control purposes (Figure 3D).

In a second series of experiments, an alternative method of NET quantification was applied which allowed for “cell free”- and “anchored”-NETs distinction by following the methodology described by Tanaka et al. (52). Overall, zymosan treatment which was used for positive control resulted in a highly significant increase of both types of NETs, i.e., “cell free”- and “anchored”-NETs in bovine PMN (*p* < 0.0001; Figures 3E–H). In addition, confrontation of PMN with *B. besnoiti* tachzoites in principle also triggered both kinds of NETs as seen for “anchored”-NETs in the rapamycin-related (*p* = 0.008; Figure 3E) and the wortmannin-related data set (*p* = 0.007; Figure 3G) and for “cell free”-NETs in the wortmannin-related dataset (*p* = 0.02; Figure 3H). Interestingly, the induction of “anchored”-NETs by parasite stages was more evident than the induction of “cell free”-NETs. In agreement with the data mentioned above, neither rapamycin nor wortmannin treatments led to altered parasite-triggered “cell free”- nor “anchored” NET formation (Figures 3E–H). Since autophagy is a very complex process, and various signaling pathways are involved in autophagosome formation, we used more pharmacological regulatory factors to check if autophagy affects NET formation via appropriate pathways. In our experimental setting, LY294002 (PI3K inhibitor) and pathenolide (NF-κB inhibitor) nor WP1130 (deubiquitinase inhibitor) treatments did not alter anchored and cell free NET formation (Figure 3I,J).

**Figure 4:**
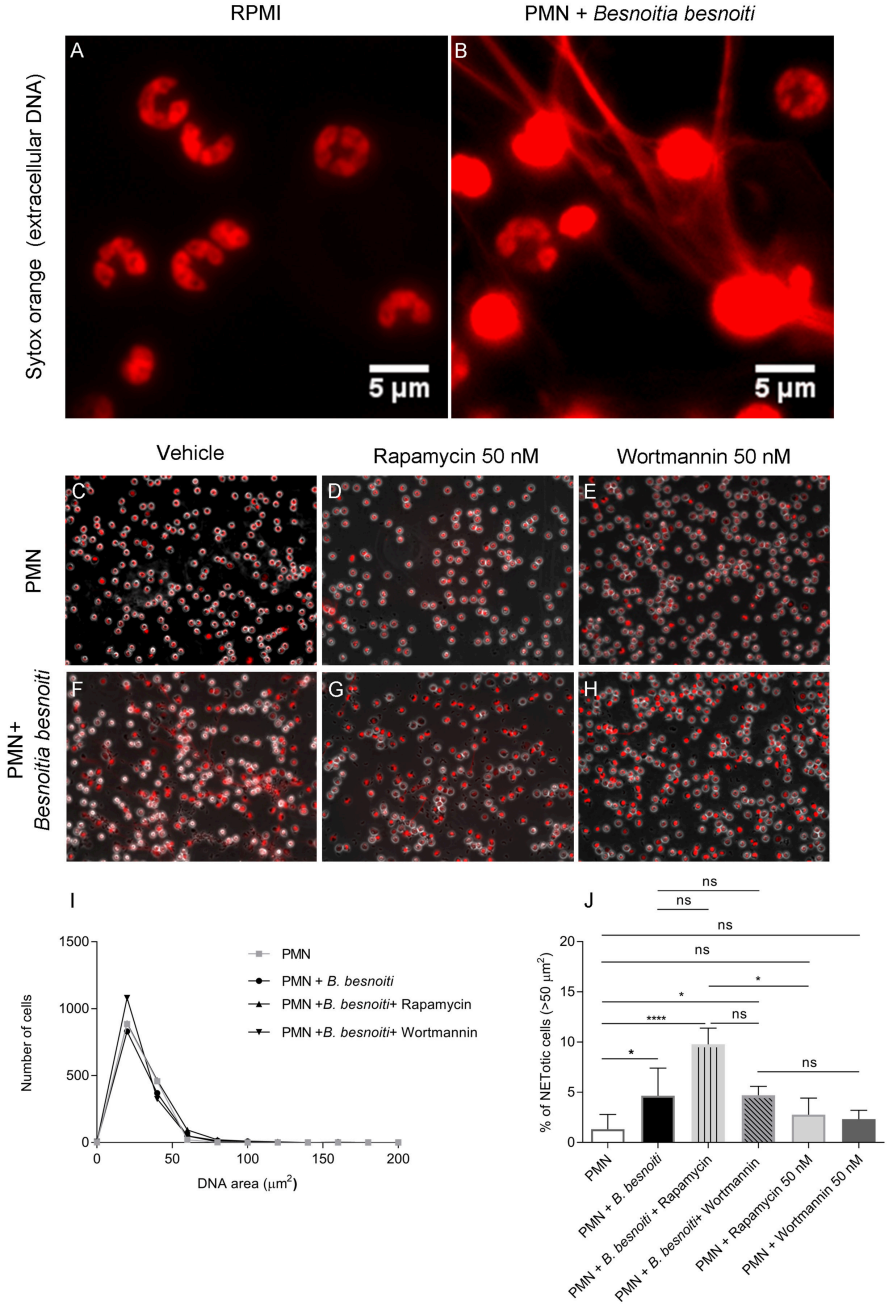
Effects of rapamycin and wortmannin treatments on *Besnoitia besnoiti*-triggered NET formation as detected by nuclear area expansion (NAE)-based quatification. Bovine PMN were incubated in media or exposed to *B. besnoiti* tachyzoites on coverslips under different experimental conditions. **(A)** PMN alone (zoom); **(B)** PMN+*B. besnoiti* (zoom) showing PMN doing NETs and considered as positive for the cell analysis software; **(C)** PMN + rapamycin 50 nM; **(D)** PMN+*B. besnoiti* + rapamycin 50 nM; **(E)** PMN + wortmannin 50 nM; **(F)** PMN+*B. besnoiti* **(G)** PMN+*B. besnoiti* + rapamycin 50 nM; **(H)** PMN+*B. besnoiti* + wortmannin 50 nM. After fixation, NAE of 1,200–1,500 cells per condition was analyzed by ImageJ **(I)** and the percentage of NETotic cells (NAE > 50 μm^2^) was calculated **(J)**. Statistical significance was defined by a *p*-value <0.05 (*p < 0.05, ****p < 0.0001) in Mann-Whitney test followed by a Dunn's multiple comparisons test.

In a third series of experiments, we chose to analyze parasite-triggered NET formation based on nuclear area expansion (NAE). In general, PMN undergo several stages of NET formation including NE- and MPO-dependent chromatin decondensation (54). Decondensed PMN nuclei are considered as a marker for early “NETotic” processes (55). In order to determine if *B. besnoiti* induces NAE in bovine PMN, as well as to estimate if rapamycin or wortmannin influences this parameter of NET formation, 1,200–1,700 cells were individually analyzed per condition (Figures 4C–H) and data illustrated via frequency histograms (Figure 4I) and percentage of cells undergoing early NET formation (Figure 4J). In agreement to data on the later phase of NET formation (Figure 3), confrontation with *B. besnoiti* tachyzoites significantly induced nuclear area expansion in a higher percentage of bovine PMN thereby indicating early NET formation processes (*p* = 0.03); illustrated in Figures 4B,F, data sets in Figures 4I,J, control condition is shown in Figures 4A,C. Interestingly, we also observed an increase of PMN populations showing NAE in case of rapamycin treatments (inducer of autophagy) of tachyzoite-exposed PMN, however, these reactions showed no significance in relation to untreated controls (PMN + *B. besnoiti*) due to the high individual variation of the donors already mentioned above (Figure 4J). Given that significant differences were indeed detected referring to rapamycin-treated tachyzoite-exposed groups and to parasite-free rapamycin controls (*p* = 0.04), an influence of rapamycin and therefore of autophagy on parasite-triggered NET formation may be stated in that sense that induction of autophagy leads to enhanced early NET formation. The fact that this effect could not be detected by the other methods of NET quantification used before (see Figure 3) may be due to the targeted early/late phase of NET formation.

### Effects of *B. besnoiti* Tachyzoite Exposure on Autophagosome Formation in PMN

**Figure 5:**
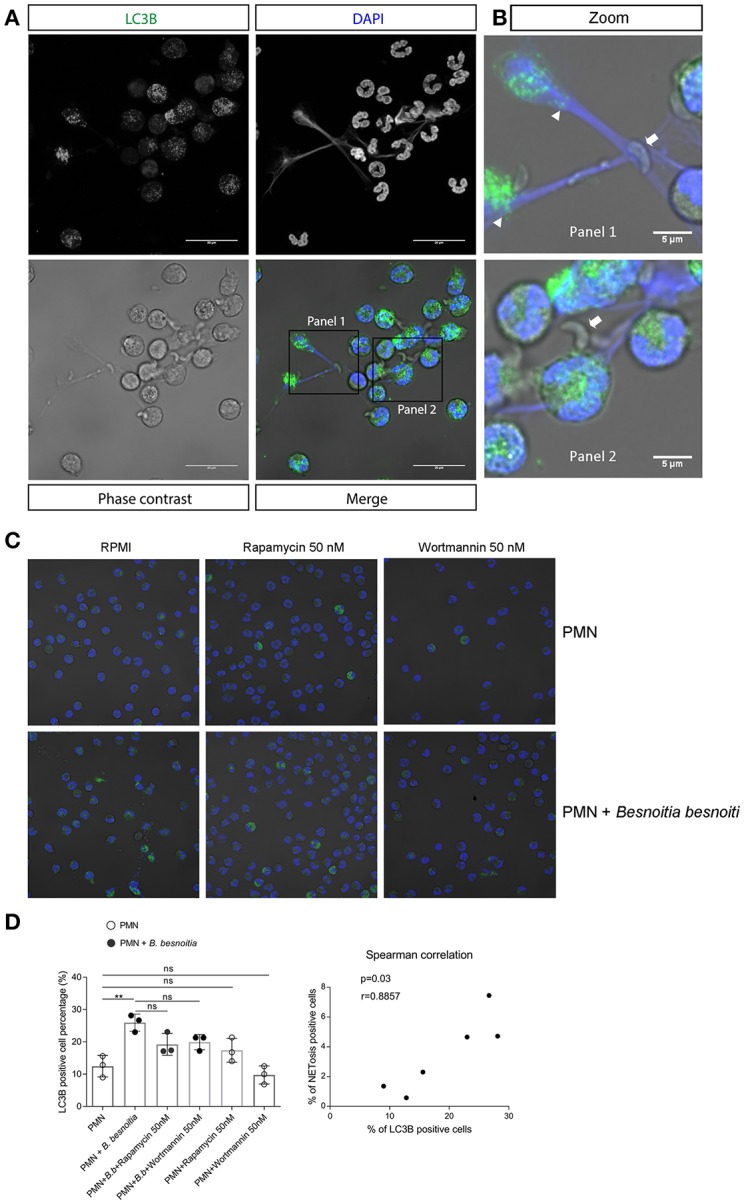
Autophagy and NET formation occurs simultaneously in *B. besnoiti*-exposed PMN. Bovine PMN were exposed to *B. besnoiti* tachyzoites for 3 h. Samples were fixed and permeabilized for LC3B-based immunostaining to determine autophagosome formation by confocal microscopy. **(A)** Panel showing the staining for LC3B (green), DAPI (blue) phase contrast (gray scale) and merge. **(B)** Zoom-in showing autophagosome formation (triangles) and NETs entrapping tachyzoites (arrows). **(C,D)** PMN were pretreated with rapamycin or wortmannin (50 nM for 30 min) and then exposed to *B. besnoitia* tachyzoites. After 3 h of incubation, the samples were stained for LC3B **(C)** and the number of autophagosome-positive cells was determined (**D**, left graph). Finally, Spearman test (**D**, graph on the right side) revealed a positive correlation (*r* = 0.89) between LC3B expression and NETotic cells. (**p < 0.01, Kruskal Wallis test, followed by Dunn's *post-hoc* test for multiple comparisons).

Given that data on NAE indicated a link between autophagy and parasite-triggered early NET formation, we additionally analyzed the effect of tachyzoite exposure on PMN-derived autophagosome formation. During autophagy, the cytosolic form of microtubule-associated protein 1A/1B-light chain 3 (LC3-I) is conjugated to phosphatidylethanolamine to form LC3-II which allows LC3 to become associated with autophagic vesicles (58). We therefore used an antibody directed against the splice variant LC3B as a marker to investigate the effect of tachyzoite exposure to PMN-derived autophagy and of autophagy on *B. besnoiti*-induced bovine NET formation. Overall, exposure to *B. besnoiti* tachyzoites led to significant autophagosome formation in exposed bovine PMN (*p* = 0.01); for illustration, see Figures 5A,B; triangles, for data see Figure 5D. Notably, cells undergoing autophagy also showed NET formation against *B. besnoiti* tachyzoites, which were firmly entrapped in DAPI-labeled chromatin structures (illustrated in Figure 5B, arrows, more images are shown in Figure S3). Thus, we analyzed LC3B expression in tachyzoites-exposed PMN and in rapamycin and wortmannin pretreated PMN (Figure 5C). The percentage of LC3B positive cells was increased in the *B. besnoitia* exposed condition but was not affected by rapamycin or wortmannin (Figure 5D). However, an increase of 8% was observed in PMA-treated PMN that were incubated previously with rapamycin Figure S1. Finally we analyzed the data by Spearman correlation test indicating a positive correlation of NET formation and autophagy in tachyzoite-exposed PMN (Figure 5D). In a different approach we evaluated autophagosome formation in PMN confronted with isolated NETs obtained as described (59). Isolated NETs failed to induce autophagosome formation. Figures and analysis are shown in Figure S2.

### *B. besnoiti* Tachyzoite Exposure Induces LC3B and p-AMPKα Protein Expression in Bovine PMN

**Figure 6:**
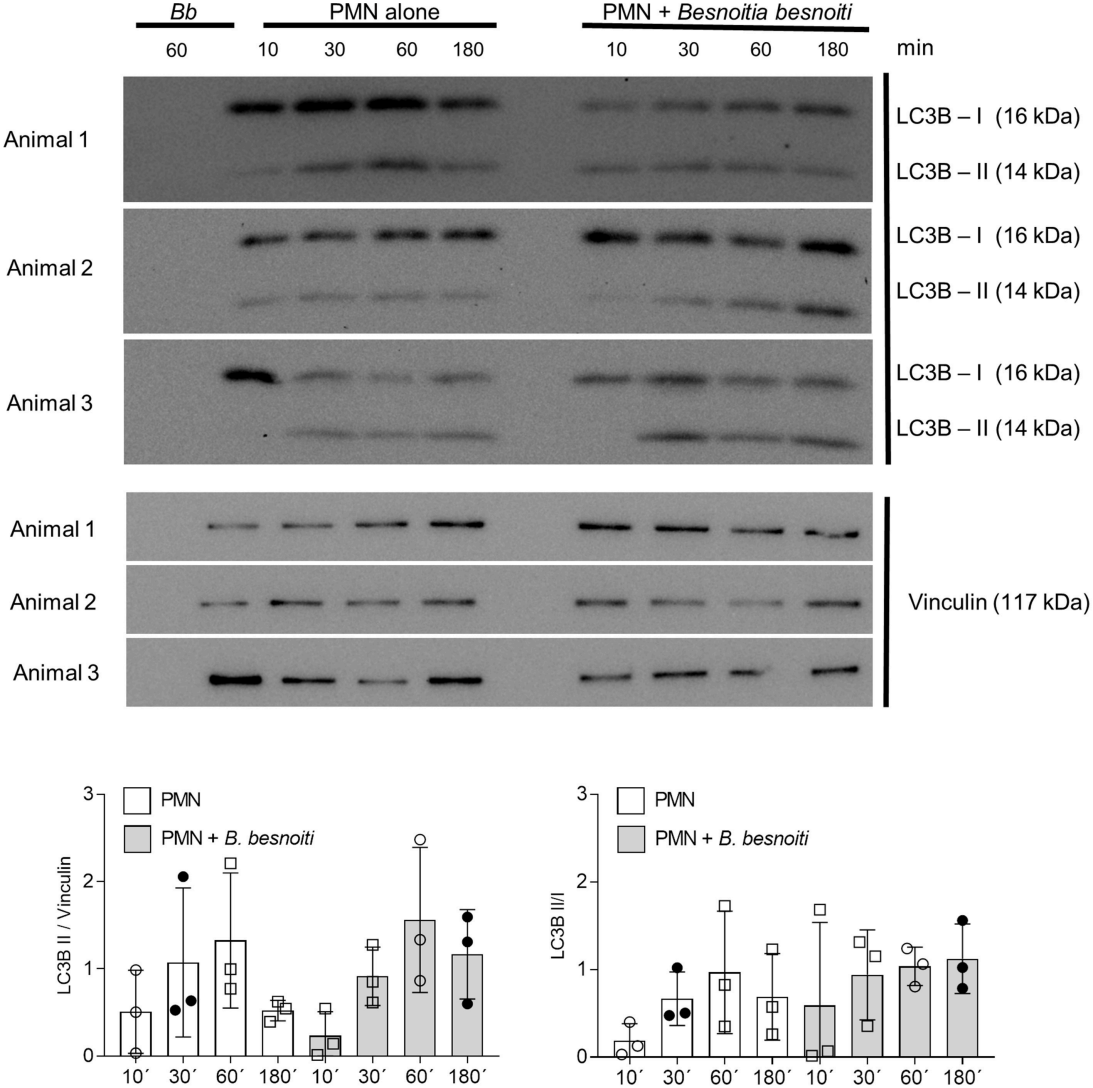
LC3B expression in *B. besnoiti* tachyzoite-exposed PMN. Protein extracts of *B. besnoiti* tachyzoite-exposed PMN (*n* = 3) were performed at different time points after exposure and analyzed for LC3B-I and LC3B-II expression by immunoblotting. The expression of vinculin was controlled as an internal reference protein.

**Figure 7:**
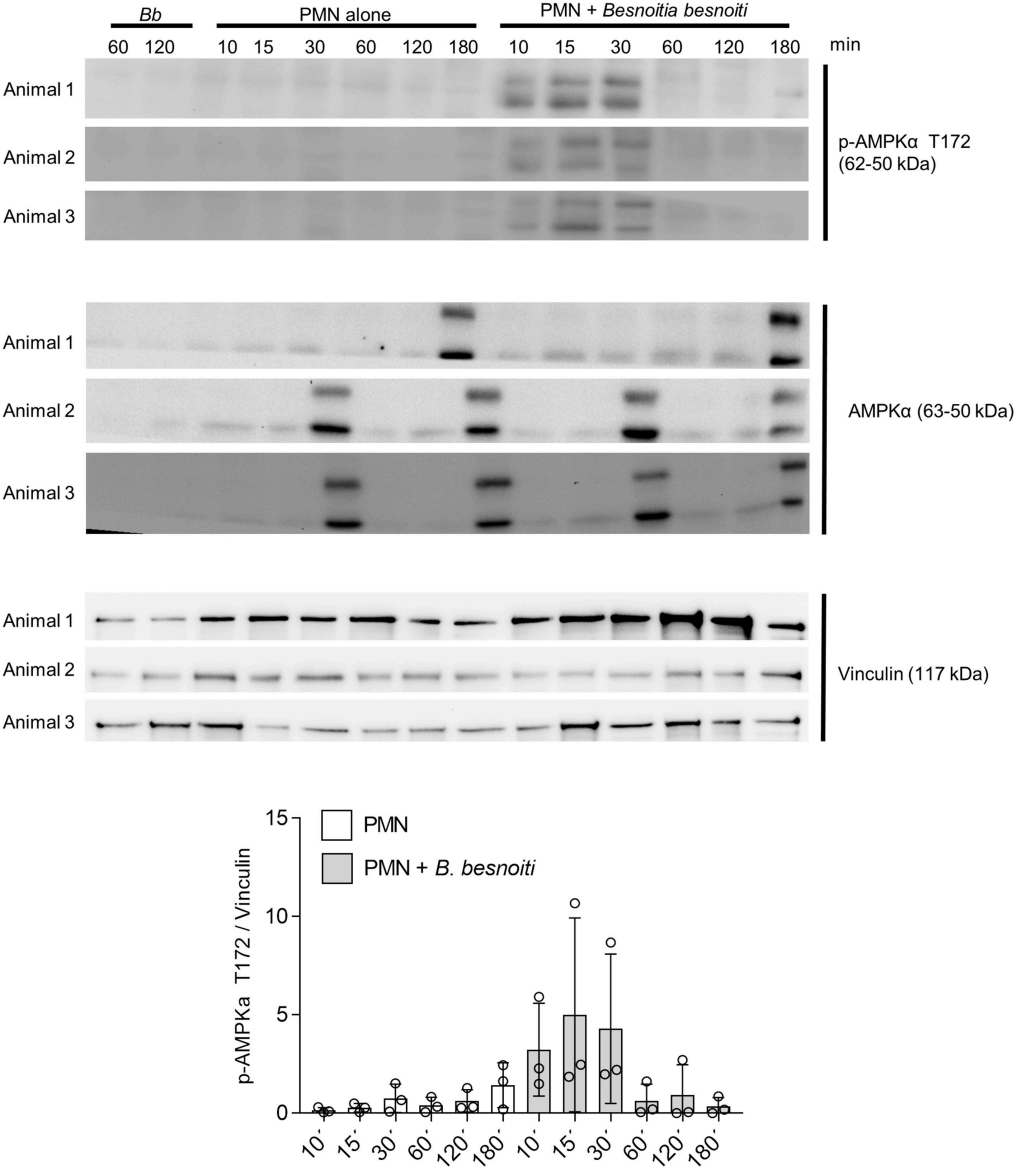
*B. besnoiti* induces the phosphorylation of AMPK in bovine PMN. Protein extracts of 5 × 10^6^ PMN confronted with 20 × 10^6^ *B. besnoiti* tachyzoites from three different donors were obtained at different time points and the kinetics were analyzed for LC3B-I and LC3B-II (upper panel) and AMPKα T172 phosphorylation (middle panel) through western blot. For AMPK, the activation of the enzyme was detected in the first 30 min of interaction (middle panel). Vinculin detection via western blot was performed for the three donors as a protein quantity loading controls.

Given that exposure to *B. besnoiti* tachyzoites induces autophagy in bovine PMN, we here analyzed the protein expression of LC3B and of phosphorylated AMPKα as a key regulator molecule of autophagy in a kinetic experiment (protein extracts of tachyzoite-exposed PMN isolated after 5, 30, 60, and 180 min of co-culture). For control reasons, protein extracts from pure *B. besnoiti* tachyzoites (three different isolates, 60 min of incubation) were included in the assays (Figure 6). Immunoblotting analyses revealed a non-statistically significant increase in LC3BII expression in *B. besnoiti* tachyzoite-confronted PMN when compared non-exposed PMN (Figure 6). Atg 5, which plays a role in the formation and elongation of autophagosomes does not show a detectable difference in protein expression when PMN were confronted with *B. besnoiti* tachyzoites (Figure S4). In addition, immunoblotting experiments revealed a very distinct expression profile on p-AMPKα. Here we used an antibody that is specific for AMPK that showed phosphorylation in the alpha subunit and thereby reflected AMPK activation. Whilst neither pure tachyzoites nor PMN alone showed any signal of this molecule, AMPKα expression was clearly induced by tachyzoite exposure of PMN thereby showing a fast response pattern with enhanced expression only within the first 30 min of contact (Figure 7, lower panel). In all samples, two distinct protein bands at the level of the expected size (~62 and 50 kDa) were observed in AMPKα-positive Immunoblots, which most probably represents parts of a cleaved form of AMPK, a common process occurring in leukocytes (60). For AMPK, a transient pattern was observed in both, *B. besnoiti* confronted and non-confronted PMN (Figure 7, middle panel) showing peaks of expression at 30 and 180 min. For comparison, p-AMPKα band densitometry was obtained and normalized by vinculin signal, reinforcing the clear effect over AMPK phosphorylation showed in immunoblots. Due to the lack of signal of AMPK in animal 3, the normally used ratio of pAMPK/AMPK was not possible to apply for all the donors; however the graph corresponding to this ratio is shown in the Figure S5.

## Discussion

The initial description of NET formation was released in 2004 and was followed by the discovery of a novel programmed cell death pathway nowadays known as ETosis (19, 20, 61). ETosis comprises a unique series of cellular events by which nuclear contents, including chromatin and histones, mix with granular/cytoplasmic components and are released from the cell to form sticky extracellular structures capable of trapping and killing microorganisms (20, 62). Meanwhile, ETosis has been implicated in diverse diseases ranging from conditions of sterile inflammation, i.e., human gout (63, 64) and bovine synovitis (65), reproduction disorders (66, 67), cancer (68, 69), and autoimmune diseases (70, 71). Since the initial report on PMN, other leukocyte types, such as monocytes, macrophages, eosinophils, basophils, and mast cells were identified to extrude ETs (72, 73).

Concerning stimuli, both chemicals and pathogens can trigger ETosis (74, 75). Several reports have demonstrated that ETosis is efficiently induced by protozoan and metazoan parasites, such as *T. gondii, E. bovis, Cryptosporidium parvum, N. caninum, Dirofilaria immitis, Haemonchus contortus*, and *Schistosoma japonicum* (18, 23, 34, 37, 76, 77). Recently, Muñoz-Caro et al. (13) confirmed *B. besnoiti* tachyzoites as potent NET inducers by quantification of extracellular DNA using Picogreen®. In the current study, we aimed to take a more detailed look on *B. besnoiti*-triggered NET formation and to dissect these immune reactions into different NET types (“anchored”/”cell-free” NETs) and time-dependent reactions (early phase of NET formation vs. finalized NETs) by using different methodical approaches. Nevertheless, as proof of principle, ETosis events were first confirmed by SEM analysis in *B. besnoiti* tachyzoites-exposed bovine PMN. Immunostaining analyses revealed that these extracellular structures were mainly composed of DNA being decorated with histones, MPO and NE, thereby confirming classical components of ETs.

As also performed in the current study and leading to the confirmation of tachyzoites as NET inducers, NET formation can be estimated by quantification of extracellular DNA using specific probes as Picogreen® (19) following DNA digestion in cell culture microplates. The advantage of this approach is obvious since several compounds can be tested in the same experiment, however it is advised by several authors that NET quantification should always be confirmed by microscopy (55, 78). In the current study we broadened the methodical panel to dissect between early events during NET formation by analyzing nuclear decondensation of PMN and late events by estimating the formation of “cell free” and “anchored” NETs. Overall, exposure of PMN to *B. besnoiti* tachyzoites led to a significant induction of nuclear decondensation and of “anchored”-NETs. Interestingly, the data were more consistent in terms of magnitude of the response in the formation of “anchored” (1.7-fold increase) than “cell free” NETs (1.4-fold increase). Thus, it appears that tachyzoites mainly induce “anchored” NETs. Of note was the inter-donor variation concerning NET formation quantification using this technique. However, this observation is in line with high inter-donor variations in terms of quantity and quality of NETs induced by soluble mediators such as PMA and A23187 (59, 79). In a further experimental approach using *B. besnoiti*-infected primary endothelium in a parallel plate flow chamber, we could confirm the formation of “anchored”-NETs under physiological flow conditions by co-localization of extracellular DNA with histones. This confirms a potential cross-talk between activated endothelium and leukocytes to be recruited to site of infection. Whether these extruded “anchored”-NETs might impact on intracellular located *B. besnoiti* tachyzoites needs further investigation. However, this observation might have implications on the outcome of cattle besnoitiosis since the tachyzoite stages are indeed infecting endothelium of vessels *in vivo* (10, 12).

Autophagy is a physiological process within the body which maintains homeostasis or normal function of cells by protein degradation and turnover of destroyed cell organelles for new cell formation after cellular stress (80). Furthermore, autophagy has been shown to play a pivotal role in regulating early innate leukocyte-associated effector mechanisms against pathogens, such as phagocytosis (81), cytokine secretion (82), and NET formation (46). In this regards, mTOR pathway plays a key role in NET formation via regulation of autophagy pathways (48). Thus, Park et al. (50) showed using a Sytox Green-related assay for NET quantification, that rapamycin pretreatments primed human PMN enhancing NET formation in response to PMA. This reaction was reversed by a panel of different autophagy inhibitors. In this set-up, rapamycin treatments alone did not influence NET formation. In our experimental set-up using *B. besnoiti* tachyzoites instead of PMA, rapamycin treatments did not influence the degree of parasite-triggered bovine NET formation when using PicoGreen-based analyses on total NETs and “anchored”/”cell-free” NETs. In addition, treatments with the autophagy-inhibitor wortmannin failed to affect parasite-triggered NET formation. This observation was complemented with the use of the PI3K inhibitor LY294002, observing a non-significant decrease of “anchored” NETs formation. Same result was observed by the use of NF-κB inhibitor. However, when estimating early NET formation via nuclear area expansion, (NAE) analysis, we found that rapamycin pretreatments indeed primed bovine PMN for enhanced NET formation in response to tachyzoites. The discrepancy between the different methods of NET detection may be explained by two factors: first, autophagy appears to precede NET formation and may therefore rather be linked to early NET formation that to late NET-related effects, and secondly, the NAE-based assay appeared more sensitive for the detection of tachyzoite-triggered NET formation and may therefore have produced an improved resolution of the data. We therefore assume that early tachyzoite-triggered NET formation is indeed linked to autophagy in bovine PMN. Furthermore, the fact that formation of LC3B-positive autophagosomes was observed in bovine PMN while casting NETs supported the potential role of autophagy in PMN-derived responses against tachyzoite stages.

Autophagosomes are double-membraned vesicles formed during autophagy, which represent characteristic markers of autophagy. LC3 is a small, soluble protein, which is distributed ubiquitously in mammalian tissues and in cultured cells. During autophagy, LC3-I (a cytosolic form of LC3) is conjugated to phosphatidylethanolamine to form LC3-II, which is then recruited to autophagosomal membranes (58). Therefore, LC3-II is widely used as a marker for the microscopic detection of intracellular autophagosomes. The LC3 gene family comprises three members, LC3A, LC3B and LC3C, and LC3B represents the most used endogenous autophagic marker (58). Certain studies have revealed that autophagy is required for NET formation (46, 83) and that autophagy induction triggers NET formation (45, 46, 83). To detect autophagy in *B. besnoiti* tachyzoite-exposed PMN as a matter of principle, autophagosome formation was visualized by LC3B-based immunostaining. Confocal microscopy clearly showed that confrontation of PMN with *B. besnoiti* tachyzoites indeed caused a significant increase of autophagosome formation. As a highly interesting finding, we additionally observed that autophagic PMN also performed NET formation. However, neither rapamycin nor wortmannin pre-treatments had any influence on PMN-derived autophagosome formation, reinforcing the observation that these processes were mTOR-independent. Our results are in line with those obtained in human PMN, where rapamycin by its own is not able to induce autophagy but increases the autophagosome formation induced by PMA (48). In line, mTOR-independent induction of autophagy was also reported in a distinct population of PMN from sepsis patients which showed increased PMA-triggered NET formation activity (45).

AMPKα is a key metabolic master regulator in eukaryotes with high impact on several important cellular mechanisms. AMPKα activation is initiated by changes in the metabolic status which result from inhibition of ATP generation during hypoxia, glucose deprivation and increased ATP consumption (84). Previous observations in PMN showed that AMPK activation decreased PMA-induced ROS production in human PMN (49), but enhanced PMN chemotaxis, bacterial killing, and phagocytosis (50). Moreover, AMPK promotes autophagy by directly activating Ulk1 which is a mTOR downstream enzyme during autophagosome formation (85). On the other hand, inhibition of AMPK in mice model induced histone 3 secretion, suggesting that AMPK activation contributed to murine NET formation (86). Since autophagy is a complex process and could be initiated via various signaling pathways, we tried to check if AMPK pathway is involved in *B. besnoiti* tachyzoite-induced autophagy. Our current data show that confrontation of PMN with *B. besnoiti* tachyzoites clearly induced AMPKα activation in a time-dependent manner. Thus, AMPKα phosphorylation was immediately induced from the very beginning of parasite-PMN interactions until 30 min of co-culture. So far, it is unclear if enhanced AMPKα activation is linked to autophagy or NET formation or both in tachyzoite-exposed neutrophils, but this will be a matter for further research.

## Ethics Statement

This study was conducted in accordance to Justus Liebig University Giessen Animal Care Committee Guidelines. Protocols were approved by Ethic Commission for Experimental Animal Studies of Federal State of Hesse (Regierungspräsidium Giessen; A9/2012; JLU-No.521_AZ) and in accordance to European Animal Welfare Legislation: ART13TFEU and current applicable German Animal Protection Laws.

## Author Contributions

CH, AT, and IC: designed the project and experiments. EZ: carried out most of the experiments. TM-C: under flow experiments, SEM. UG: SEM and confocal microscope. ZV: LC3B confocal microscopy and Western blots. IC, CH, and AT: prepared the manuscript. IC, ZV, and EZ: prepared the figures. All authors reviewed the manuscript.

### Conflict of Interest Statement

The authors declare that the research was conducted in the absence of any commercial or financial relationships that could be construed as a potential conflict of interest.

## Supplementary Material

Figure S1 Autophagy induction in bovine PMN. Bovine PMN were treated with PMA (20 nM) and rapamycin (100 nM) for 1 h, and bovine PMN treated with media was used as control. Samples were fixed and permeabilized with pure cold methanol for LC3B-based immunostaining to determine autophagosome formation by confocal microscopy. **(A,B)** showing merged images with the staining for LC3B (green), DAPI (blue) in the control group. **(C,D)** showing merged images with the staining for LC3B (green), DAPI (blue) in the treated group. The right graph shows the percentage of autophagosome-positive cells **(E)**.

Figure S2 Isolated NETs fails to induce autophagosome formation in bovine PMN. Isolated NETs from *B. besnoiti*-confronted PMN were isolated as described by Barrientos (59). Bovine PMN were treated with isolated NETs for 1 h, and bovine PMN treated with media was used as control. Samples were fixed and permeabilized with pure cold methanol for LC3B-based immunostaining to determine autophagosome formation by confocal microscopy. **(A,B)** showing merged images with the staining for LC3B (green), DAPI (blue) in the control group. **(C,D)** showing merged images with the staining for LC3B (green), DAPI (blue) in the treated group. The right graph shows the percentage of autophagosome-positive cells **(E)**.

Figure S3 Autophagy and NET formation occurs simultaneously in *B. besnoitia*-exposed PMN. Bovine PMN were exposed to *B. besnoitia* tachyzoites for 3 h. Samples were fixed and permeabilized for LC3B-based immunostaining to determine autophagosome formation by confocal microscopy. **(A–F)** control group: **(A,C,E)** phase contrast, **(B,D,F)** merged images; **(G–L)** PMN+B. besnoiti group: **(G,I,K)** phase contrast **(H,J,L)** merged images. Blue: DNA staining with DAPI, green: autophagosomes staining with L3CB antibody.

Figure S4 Atg5 protein expression in *B. besnoitia*-confronted PMN.

Figure S5 Densitometry quantification of p-AMPKa T127/AMPK.

Figure S6 NET formation in control bovine PMN (1/2). Analysis at the same time-point of the experiments performed with *B. besnoitia* tachyzoites. **(A)** Phase contrast image; **(B)** DNA staining: Sytox Orange; **(C)** histone (H11-4) staining; **(D)** neutrophil elastase (NE) staining; **(E)** Merged image of **B–D** and **(F)** Merged image of all channels **(A–D)**.

Figure S7 NET formation in control bovine PMN (2/2). Analysis at the same time-point of the experiments performed with *B. besnoitia* tachyzoites. **(A)** Phase contrast image; **(B)** DNA staining: Sytox Orange; **(C)** histone (H11-4) staining; **(D)** neutrophil elastase (NE) staining; **(E)** Merged image of **B–D** and **(F)** Merged image of all channels **(A–D)**.

## References

[1] Basso W Lesser M Grimm F Hilbe M Sydler T Trösch L. Bovine besnoitiosis in Switzerland: imported cases and local transmission. Vet Parasitol. (2013) 198:265–73. 10.1016/j.vetpar.2013.09.013 24120579

[2] Cortes HCE Reis Y Waap H Vidal R Soares H Marques I. Isolation of *Besnoitia besnoiti* from infected cattle in Portugal. Vet Parasitol. (2006) 141:226–33. 10.1016/j.vetpar.2006.05.022 16822614

[3] Fernández-García A Risco-Castillo V Pedraza-Díaz S Aguado-Martínez A Alvarez-García G Gómez-Bautista M. First isolation of *Besnoitia besnoiti* from a chronically infected cow in Spain. J Parasitol. (2009) 95:474–6. 10.1645/GE-1772.1 18803440

[4] Gentile A Militerno G Schares G Nanni A Testoni S Bassi P. Evidence for bovine besnoitiosis being endemic in Italy–first *in vitro* isolation of *Besnoitia besnoiti* from cattle born in Italy. Vet Parasitol. (2012) 184:108–15. 10.1016/j.vetpar.2011.09.014 21978744

[5] Gollnick NS Gentile A Schares G. Diagnosis of bovine besnoitiosis in a bull born in Italy. Vet Rec. (2010) 166:599. 10.1136/vr.c2314 20453244

[6] Hornok S Fedák A Baska F Hofmann-Lehmann R Basso W. Bovine besnoitiosis emerging in Central-Eastern Europe, Hungary. Parasit Vectors. (2014) 7:20. 10.1186/1756-3305-7-20 24410743 PMC3895772

[7] Jacquiet P Liénard E Franc M. Bovine besnoitiosis: epidemiological and clinical aspects. Vet Parasitol. (2010) 174:30–6. 10.1016/j.vetpar.2010.08.013 20850933

[8] Rinaldi L Maurelli MP Musella V Bosco A Cortes H Cringoli G. First cross-sectional serological survey on *Besnoitia besnoiti* in cattle in Italy. Parasitol Res. (2013) 112:1805–7. 10.1007/s00436-012-3241-y 23274487

[9] Schares G Basso W Majzoub M Cortes HCE Rostaher A Selmair J. First *in vitro* isolation of *Besnoitia besnoiti* from chronically infected cattle in Germany. Vet Parasitol. (2009) 163:315–22. 10.1016/j.vetpar.2009.04.033 19477592

[10] Álvarez-García G Frey CF Mora LMO Schares G. A century of bovine besnoitiosis: an unknown disease re-emerging in Europe. Trends Parasitol. (2013) 29:407–15. 10.1016/j.pt.2013.06.002 23830145

[11] Alvarez-García G García-Lunar P Gutiérrez-Expósito D Shkap V Ortega-Mora LM. Dynamics of *Besnoitia besnoiti* infection in cattle. Parasitology. (2014) 141:1419–35. 10.1017/S0031182014000729 24871877

[12] Maksimov P Hermosilla C Kleinertz S Hirzmann J Taubert A. *Besnoitia besnoiti* infections activate primary bovine endothelial cells and promote PMN adhesion and NET formation under physiological flow condition. Parasitol Res. (2016) 115:1991–2001. 10.1007/s00436-016-4941-5 26847631

[13] Muñoz-Caro T Hermosilla C Silva LMR Cortes H Taubert A. Neutrophil extracellular traps as innate immune reaction against the emerging apicomplexan parasite *Besnoitia besnoiti*. PLoS ONE. (2014) 9:e91415. 10.1371/journal.pone.0091415 24618849 PMC3950022

[14] Behrendt JH Hermosilla C Hardt M Failing K Zahner H Taubert A. PMN-mediated immune reactions against *Eimeria bovis*. Vet Parasitol. (2008) 151:97–109. 10.1016/j.vetpar.2007.11.013 18155359

[15] Taubert A Behrendt JH Sühwold A Zahner H Hermosilla C. Monocyte- and macrophage-mediated immune reactions against *Eimeria bovis*. Vet Parasitol. (2009) 164:141–53. 10.1016/j.vetpar.2009.06.003 19559532

[16] Behrendt JH Ruiz A Zahner H Taubert A Hermosilla C. Neutrophil extracellular trap formation as innate immune reactions against the apicomplexan parasite *Eimeria bovis*. Vet Immunol Immunopathol. (2010) 133:1–8. 10.1016/j.vetimm.2009.06.012 19625090

[17] Silva LMR Muñoz Caro T Gerstberger R Vila-Viçosa MJM Cortes HCE Hermosilla C. The apicomplexan parasite *Eimeria arloingi* induces caprine neutrophil extracellular traps. Parasitol Res. (2014) 113:2797–807. 10.1007/s00436-014-3939-0 24849865

[18] Villagra-Blanco R Silva LMR Muñoz-Caro T Yang Z Li J. Bovine polymorphonuclear neutrophils cast neutrophil extracellular traps against the abortive parasite *Neospora caninum*. Front Immunol. (2017) 8:606. 10.3389/fimmu.2017.00606 28611772 PMC5447047

[19] Fuchs TA Abed U Goosmann C Hurwitz R Schulze I Wahn V. Novel cell death program leads to neutrophil extracellular traps. J Cell Biol. (2007) 176:231–41. 10.1083/jcb.200606027 17210947 PMC2063942

[20] Brinkmann V Zychlinsky A. Neutrophil extracellular traps: is immunity the second function of chromatin? J Cell Biol. (2012) 198:773–83. 10.1083/jcb.201203170 22945932 PMC3432757

[21] Hahn S Giaglis S Chowdhury CS Chowdury CS Hösli I Hasler P. Modulation of neutrophil NETosis: interplay between infectious agents and underlying host physiology. Semin Immunopathol. (2013) 35:439–53. 10.1007/s00281-013-0380-x 23649713 PMC3685704

[22] Parker H Winterbourn CC. Reactive oxidants and myeloperoxidase and their involvement in neutrophil extracellular traps. Front Immunol. (2013) 3:424. 10.3389/fimmu.2012.00424 23346086 PMC3549523

[23] Muñoz-Caro T Conejeros I Zhou E Pikhovych A Gärtner U Hermosilla C. *Dirofilaria immitis* microfilariae and third-stage larvae induce canine NETosis resulting in different types of neutrophil extracellular traps. Front Immunol. (2018) 9:968. 10.3389/fimmu.2018.00968 29867950 PMC5951940

[24] Nathan C. Neutrophils and immunity: challenges and opportunities. Nat Rev Immunol. (2006) 6:173–82. 10.1038/nri1785 16498448

[25] Silva LMR Muñoz-Caro T Burgos RA Hidalgo MA Taubert A Hermosilla C. Far beyond phagocytosis: phagocyte-derived extracellular traps act efficiently against protozoan parasites *in vitro* and *in vivo*. Mediators Inflamm. (2016) 2016:1–13. 10.1155/2016/5898074 27445437 PMC4944069

[26] Hakkim A Fuchs TA Martinez NE Hess S Prinz H Zychlinsky A. Activation of the Raf-MEK-ERK pathway is required for neutrophil extracellular trap formation. Nat Chem Biol. (2011) 7:75–7. 10.1038/nchembio.496 21170021

[27] Wartha F Henriques-Normark B. ETosis: a novel cell death pathway. Sci Signal. (2008) 1:pe25. 10.1126/stke.121pe25 18506034

[28] Douda DN Khan MA Grasemann H Palaniyar N. SK3 channel and mitochondrial ROS mediate NADPH oxidase-independent NETosis induced by calcium influx. Proc Natl Acad Sci USA. (2015) 112:2817–22. 10.1073/pnas.1414055112 25730848 PMC4352781

[29] Khan MA Palaniyar N. Transcriptional firing helps to drive NETosis. Sci Rep. (2017) 7:41749. 10.1038/srep41749 28176807 PMC5296899

[30] Yipp BG Petri B Salina D Jenne CN Scott BNV Zbytnuik LD. Infection-induced NETosis is a dynamic process involving neutrophil multitasking *in vivo*. Nat Med. (2012) 18:1386–93. 10.1038/nm.2847 22922410 PMC4529131

[31] Yousefi S Mihalache C Kozlowski E Schmid I Simon HU. Viable neutrophils release mitochondrial DNA to form neutrophil extracellular traps. Cell Death Differ. (2009) 16:1438–44. 10.1038/cdd.2009.96 19609275

[32] Baker VS Imade GE Molta NB Tawde P Pam SD Obadofin MO. Cytokine-associated neutrophil extracellular traps and antinuclear antibodies in *Plasmodium falciparum* infected children under six years of age. Malar J. (2008) 7:41. 10.1186/1475-2875-7-41 18312656 PMC2275287

[33] Muñoz-Caro T Mena Huertas SJ Conejeros I Alarcón P Hidalgo MA Burgos RA. *Eimeria bovis*-triggered neutrophil extracellular trap formation is CD11b-, ERK 1/2-, p38 MAP kinase- and SOCE-dependent. Vet Res. (2015) 46:23. 10.1186/s13567-015-0155-6 25885264 PMC4349228

[34] Abi ADS Lin C Ball CJ King MR Duhamel GE Denkers EY *Toxoplasma gondii* triggers release of human and mouse neutrophil extracellular traps. Infect Immun. (2012) 80:768–77. 10.1128/IAI.05730-11 22104111 PMC3264325

[35] Reichel M Muñoz-Caro T Sanchez Contreras G Rubio García A Magdowski G Gärtner U. Harbour seal (Phoca vitulina) PMN and monocytes release extracellular traps to capture the apicomplexan parasite *Toxoplasma gondii*. Dev Compar Immunol. (2015) 50:106–15. 10.1016/j.dci.2015.02.002 25681075

[36] Yildiz K Gokpinar S Gazyagci AN Babur C Sursal N Azkur AK. Role of NETs in the difference in host susceptibility to *Toxoplasma gondii* between sheep and cattle. Vet Immunol Immunopathol. (2017) 189:1–10. 10.1016/j.vetimm.2017.05.005 28669381

[37] Muñoz-Caro T Lendner M Daugschies A Hermosilla C Taubert A. NADPH oxidase, MPO, NE, ERK1/2, p38 MAPK and Ca2+ influx are essential for *Cryptosporidium parvum*-induced NET formation. Dev Comp Immunol. (2015) 52:245–54. 10.1016/j.dci.2015.05.007 26026247

[38] Villagra-Blanco R Silva LMR Gärtner U Wagner H Failing K. Molecular analyses on *Neospora caninum* -triggered NETosis in the caprine system. Dev Compar Immunol. (2017) 72:119–27. 10.1016/j.dci.2017.02.020 28254622

[39] Wei Z Hermosilla C Taubert A He X Wang X Gong P. Canine neutrophil extracellular traps release induced by the apicomplexan parasite *Neospora caninum in vitro*. Front Immunol. (2016) 7:436. 10.3389/fimmu.2016.00436 27843440 PMC5086672

[40] Sousa-Rocha D Thomaz-Tobias M Diniz LFA Souza PSS Pinge-Filho P Toledo KA. *Trypanosoma cruzi* and its soluble antigens induce NET release by stimulating toll-like receptors. PLoS ONE. (2015) 10:e0139569. 10.1371/journal.pone.0139569 26431537 PMC4591979

[41] Ventura-Juarez J Campos-Esparza M Pacheco-Yepez J López-Blanco JA Adabache-Ortíz A Silva-Briano M. *Entamoeba histolytica* induces human neutrophils to form NETs. Parasite Immunol. (2016) 38:503–9. 10.1111/pim.12332 27138813

[42] Skendros P Mitroulis I Ritis K. Autophagy in neutrophils: from granulopoiesis to neutrophil extracellular traps. Front Cell Dev Biol. (2018) 6:109. 10.3389/fcell.2018.00109 30234114 PMC6131573

[43] Maugeri N Campana L Gavina M Covino C De Metrio M Panciroli C. Activated platelets present high mobility group box 1 to neutrophils, inducing autophagy and promoting the extrusion of neutrophil extracellular traps. J Thromb Haemost. (2014) 12:2074–88. 10.1111/jth.12710 25163512

[44] Mitroulis I Kourtzelis I Kambas K Rafail S Chrysanthopoulou A Speletas M. Regulation of the autophagic machinery in human neutrophils. Eur J Immunol. (2010) 40:1461–72. 10.1002/eji.200940025 20162553

[45] Park SY Shrestha S Youn Y-J Kim J-K Kim S-Y Kim HJ. Autophagy primes neutrophils for neutrophil extracellular trap formation during sepsis. Am J Respir Crit Care Med. (2017) 196:577–89. 10.1164/rccm.201603-0596OC 28358992

[46] Remijsen Q Vanden Berghe T Wirawan E Asselbergh B Parthoens E De Rycke R. Neutrophil extracellular trap cell death requires both autophagy and superoxide generation. Cell Res. (2011) 21:290–304. 10.1038/cr.2010.150 21060338 PMC3193439

[47] Laplante M Sabatini DM. mTOR signaling in growth control and disease. Cell. (2012) 149:274–93. 10.1016/j.cell.2012.03.017 22500797 PMC3331679

[48] Itakura A McCarty OJT. Pivotal role for the mTOR pathway in the formation of neutrophil extracellular traps via regulation of autophagy. Am J Physiol Cell Physiol. (2013) 305:C348–54. 10.1152/ajpcell.00108.2013 23720022 PMC3742850

[49] Alba G El Bekay R Alvarez-Maqueda M Chacón P Vega A Monteseirín J. Stimulators of AMP-activated protein kinase inhibit the respiratory burst in human neutrophils. FEBS Lett. (2004) 573:219–25. 10.1016/j.febslet.2004.07.077 15328001

[50] Park DW Jiang S Tadie J-M Stigler WS Gao Y Deshane J. Activation of AMPK enhances neutrophil chemotaxis and bacterial killing. Mol Med. (2013) 19:387–98. 10.2119/molmed.2013.00065 24091934 PMC3883969

[51] Taubert A Zahner H Hermosilla C. Dynamics of transcription of immunomodulatory genes in endothelial cells infected with different coccidian parasites. Vet Parasitol. (2006) 142:214–22. 10.1016/j.vetpar.2006.07.021 16930845

[52] Tanaka K Koike Y Shimura T Okigami M Ide S Toiyama Y. *In vivo* characterization of neutrophil extracellular traps in various organs of a murine sepsis model. PLoS ONE. (2014) 9:e111888. 10.1371/journal.pone.0111888 25372699 PMC4221155

[53] Lawrence MB Springer TA. Leukocytes roll on a selectin at physiologic flow rates: distinction from and prerequisite for adhesion through integrins. Cell. (1991) 65:859–73. 10.1016/0092-8674(91)90393-D 1710173

[54] Papayannopoulos V Metzler KD Hakkim A Zychlinsky A. Neutrophil elastase and myeloperoxidase regulate the formation of neutrophil extracellular traps. J Cell Biol. (2010) 191:677–91. 10.1083/jcb.201006052 20974816 PMC3003309

[55] Gonzalez AS Bardoel BW Harbort CJ Zychlinsky A. Induction and quantification of neutrophil extracellular traps. Methods Mol Biol. (2014) 1124:307–18. 10.1007/978-1-62703-845-4_20 24504961

[56] Karim MR Kanazawa T Daigaku Y Fujimura S Miotto G Kadowaki M. Cytosolic LC3 ratio as a sensitive index of macroautophagy in isolated rat hepatocytes and H4-II-E cells. Autophagy. (2007) 3:553–60. 10.4161/auto.4615 17617739

[57] Blommaart EF Krause U Schellens JP Vreeling-Sindelárová H Meijer AJ. The phosphatidylinositol 3-kinase inhibitors wortmannin and LY294002 inhibit autophagy in isolated rat hepatocytes. Eur J Biochem. (1997) 243:240–6. 10.1111/j.1432-1033.1997.0240a.x 9030745

[58] Tanida I Yamaji T Ueno T Ishiura S Kominami E Hanada K. Consideration about negative controls for LC3 and expression vectors for four colored fluorescent protein-LC3 negative controls. Autophagy. (2008) 4:131–4. 10.4161/auto.5233 18000393

[59] Barrientos L Marin-Esteban V de Chaisemartin L Le-Moal VL Sandré C Bianchini E. An improved strategy to recover large fragments of functional human neutrophil extracellular traps. Front Immunol. (2013) 4:166. 10.3389/fimmu.2013.00166 23805143 PMC3690357

[60] Zhang Z Amorosa LF Coyle SM Macor MA Lubitz SE Carson JL. Proteolytic cleavage of AMPKα and intracellular MMP9 expression are both required for TLR4-mediated mTORC1 activation and HIF-1α expression in leukocytes. J Immunol. (2015) 195:2452–60. 10.4049/jimmunol.1500944 26232429 PMC4546925

[61] Brinkmann V. Neutrophil extracellular traps kill bacteria. Science. (2004) 303:1532–5. 10.1126/science.1092385 15001782

[62] Doster RS Rogers LM Gaddy JA Aronoff DM. Macrophage extracellular traps: a scoping review. J Innate Immun. (2018) 10:3–13. 10.1159/000480373 28988241 PMC6757166

[63] Chatfield SM Grebe K Whitehead LW Rogers KL Nebl T Murphy JM. Monosodium urate crystals generate nuclease-resistant neutrophil extracellular traps via a distinct molecular pathway. J Immunol. (2018) 200:1802–16. 10.4049/jimmunol.1701382 29367211

[64] Mitroulis I Kambas K Chrysanthopoulou A Skendros P Apostolidou E Kourtzelis I. (2011). Neutrophil extracellular trap formation is associated with IL-1β and autophagy-related signaling in gout. PLoS ONE. 6:e29318. 10.1371/journal.pone.0029318 22195044 PMC3241704

[65] Alarcón P Manosalva C Conejeros I Carretta MD Muñoz-Caro T Silva LMR. d(–) lactic acid-induced adhesion of bovine neutrophils onto endothelial cells is dependent on neutrophils extracellular traps formation and CD11b expression. Front Immunol. (2017) 8:975. 10.3389/fimmu.2017.00975 28861083 PMC5559443

[66] Gupta AK Hasler P Holzgreve W Gebhardt S Hahn S. Induction of neutrophil extracellular DNA lattices by placental microparticles and IL-8 and their presence in preeclampsia. Hum Immunol. (2005) 66:1146–54. 10.1016/j.humimm.2005.11.003 16571415

[67] Zambrano F Carrau T Gärtner U Seipp A Taubert A Felmer R. Leukocytes coincubated with human sperm trigger classic neutrophil extracellular traps formation, reducing sperm motility. Fertil Steril. (2016) 106:1053–60.e1. 10.1016/j.fertnstert.2016.06.005 27344301

[68] Olsson A-K Cedervall J. NETosis in cancer – platelet–neutrophil crosstalk promotes tumor-associated pathology. Front Immunol. (2016) 7:373. 10.3389/fimmu.2016.00373 27708646 PMC5030622

[69] Thålin C Lundström S Seignez C Daleskog M Lundström A Henriksson P. Citrullinated histone H3 as a novel prognostic blood marker in patients with advanced cancer. PLoS ONE. (2018) 13:e0191231. 10.1371/journal.pone.0191231 29324871 PMC5764486

[70] Carmona-Rivera C Purmalek MM Moore E Waldman M Walter PJ Garraffo HM. A role for muscarinic receptors in neutrophil extracellular trap formation and levamisole-induced autoimmunity. JCI Insight. (2017) 2:e89780. 10.1172/jci.insight.89780 28194438 PMC5291726

[71] Lood C Blanco LP Purmalek MM Carmona-Rivera C De Ravin SS Smith CK. Neutrophil extracellular traps enriched in oxidized mitochondrial DNA are interferogenic and contribute to lupus-like disease. Nat Med. (2016) 22:146–53. 10.1038/nm.4027 26779811 PMC4742415

[72] Chow OA von Köckritz-Blickwede M Bright AT Hensler ME Zinkernagel AS Cogen AL. Statins enhance formation of phagocyte extracellular traps. Cell Host Microbe. (2010) 8:445–54. 10.1016/j.chom.2010.10.005 21075355 PMC3008410

[73] von Köckritz-Blickwede M Goldmann O Thulin P Heinemann K Norrby-Teglund A Rohde M Phagocytosis-independent antimicrobial activity of mast cells by means of extracellular trap formation. Blood. (2008) 111:3070–80. 10.1182/blood-2007-07-104018 18182576

[74] Hoppenbrouwers T Autar ASA Sultan AR Abraham TE van Cappellen WA Houtsmuller AB. *In vitro* induction of NETosis: comprehensive live imaging comparison and systematic review. PLoS ONE. (2017) 12:e0176472. 10.1371/journal.pone.0176472 28486563 PMC5423591

[75] Kenny EF Herzig A Krüger R Muth A Mondal S Thompson PR. Diverse stimuli engage different neutrophil extracellular trap pathways. Elife. (2017) 6:e24437 10.7554/eLife.24437 28574339 PMC5496738

[76] Chuah C Jones MK Burke ML McManus DP Owen HC Gobert GN. Defining a pro-inflammatory neutrophil phenotype in response to schistosome eggs. Cell Microbiol. (2014) 16:1666–77. 10.1111/cmi.12316 24898449

[77] Muñoz-Caro T Rubio RMC Silva LMR Magdowski G Gärtner U McNeilly TN. Leucocyte-derived extracellular trap formation significantly contributes to Haemonchus contortus larval entrapment. Parasit Vectors. (2015) 8:607. 10.1186/s13071-015-1219-1 26610335 PMC4661960

[78] von Köckritz-Blickwede M Chow O Ghochani M Nizet V 7 - visualization and functional evaluation of phagocyte extracellular traps. In: Kabelitz D Kaufmann SHE, editors. Methods in Microbiology, Immunology of Infection. Academic Press (2010). p. 139–60. 10.1016/S0580-9517(10)37007-3. Available online at: https://www.sciencedirect.com/science/article/pii/S0580951710370073

[79] Hoffmann JHO Schaekel K Gaiser MR Enk AH Hadaschik EN. Interindividual variation of NETosis in healthy donors: introduction and application of a refined method for extracellular trap quantification. Exp Dermatol. (2016) 25:895–900. 10.1111/exd.13125 27307108

[80] Levine B Kroemer G. Autophagy in the pathogenesis of disease. Cell. (2008) 132:27–42. 10.1016/j.cell.2007.12.018 18191218 PMC2696814

[81] Levine B Mizushima N Virgin HW. Autophagy in immunity and inflammation. Nature. (2011) 469:323–35. 10.1038/nature09782 21248839 PMC3131688

[82] Jones SA Mills KHG Harris J. Autophagy and inflammatory diseases. Immunol Cell Biol. (2013) 91:250–8. 10.1038/icb.2012.82 23318657

[83] Ullah I Ritchie ND Evans TJ. The interrelationship between phagocytosis, autophagy and formation of neutrophil extracellular traps following infection of human neutrophils by *Streptococcus pneumoniae*. Innate Immun. (2017) 23:413–23. 10.1177/1753425917704299 28399692 PMC5505230

[84] Zhao X Zmijewski JW Lorne E Liu G Park Y-J Tsuruta Y. Activation of AMPK attenuates neutrophil proinflammatory activity and decreases the severity of acute lung injury. Am J Physiol Lung Cell Mol Physiol. (2008) 295:L497–504. 10.1152/ajplung.90210.2008 18586954 PMC2536800

[85] Kim J Kundu M Viollet B Guan KL. AMPK and mTOR regulate autophagy through direct phosphorylation of Ulk1. Nat Cell Biol. (2011) 13:132–41. 10.1038/ncb2152 21258367 PMC3987946

[86] Jiang S Park DW Tadie J-M Gregoire M Deshane J Pittet JF. Human resistin promotes neutrophil proinflammatory activation and neutrophil extracellular trap formation and increases severity of acute lung injury. J Immunol. (2014) 192:4795–803. 10.4049/jimmunol.1302764 24719460 PMC4018664

