# Supplementary figures and images for: Simultaneous and Positively Correlated NET Formation and Autophagy in Besnoitia besnoiti Tachyzoite-Exposed Bovine Polymorphonuclear Neutrophils
Source: Front Immunol. 2019 May 22;10:1131. doi: 10.3389/fimmu.2019.01131 (PMC6540735; doi:10.3389/fimmu.2019.01131)

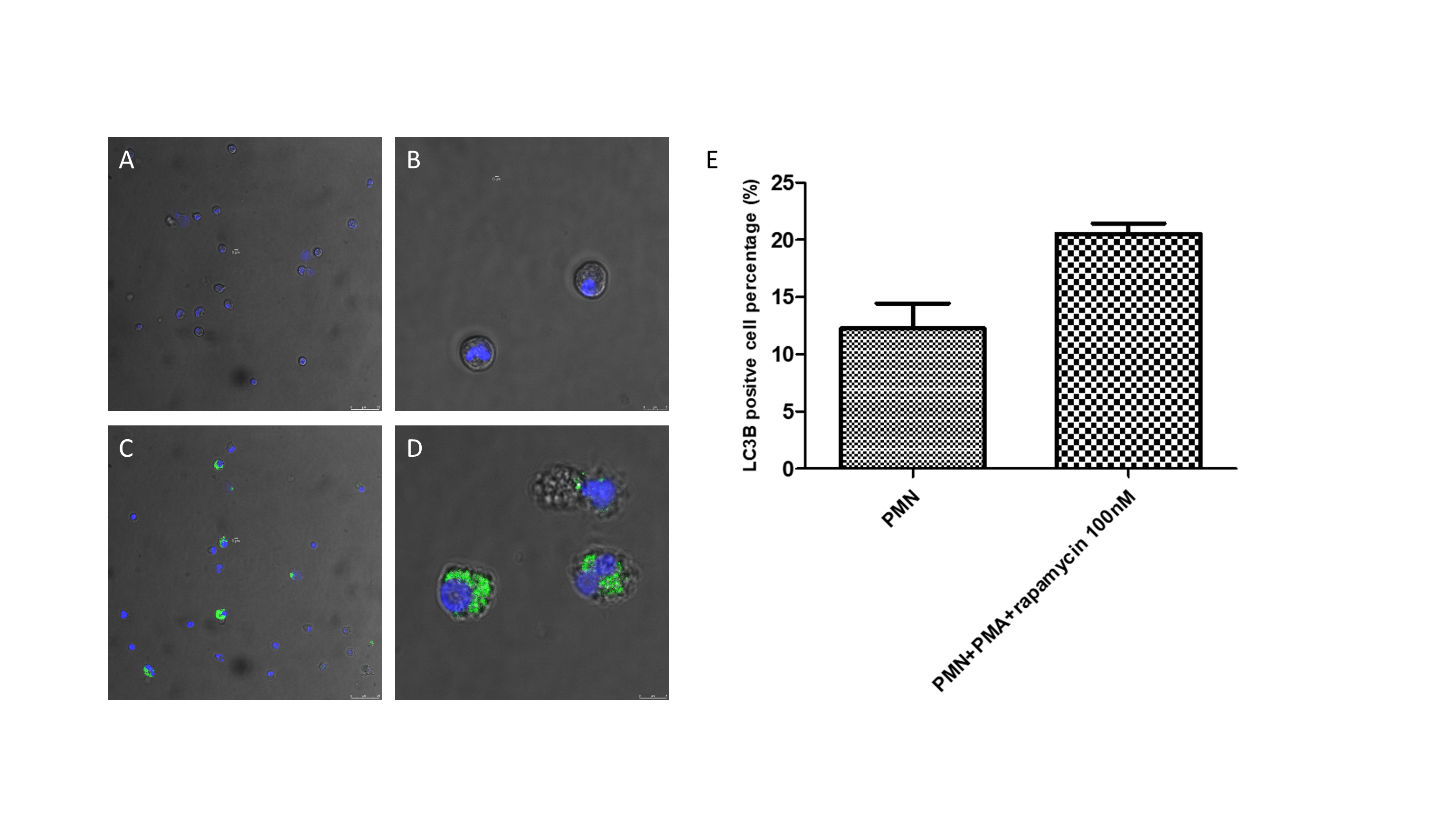

Supplement: Figure S1 — Autophagy induction in bovine PMN. Bovine PMN were treated with PMA (20 nM) and rapamycin (100 nM) for 1 h, and bovine PMN treated with media was used as control. Samples were fixed and permeabilized with pure cold methanol for LC3B-based immunostaining to determine autophagosome formation by confocal microscopy. (A,B) showing merged images with the staining for LC3B (green), DAPI (blue) in the control group. (C,D) showing merged images with the staining for LC3B (green), DAPI (blue) in the treated group. The right graph shows the percentage of autophagosome-positive cells (E). [file Image_1.TIF]

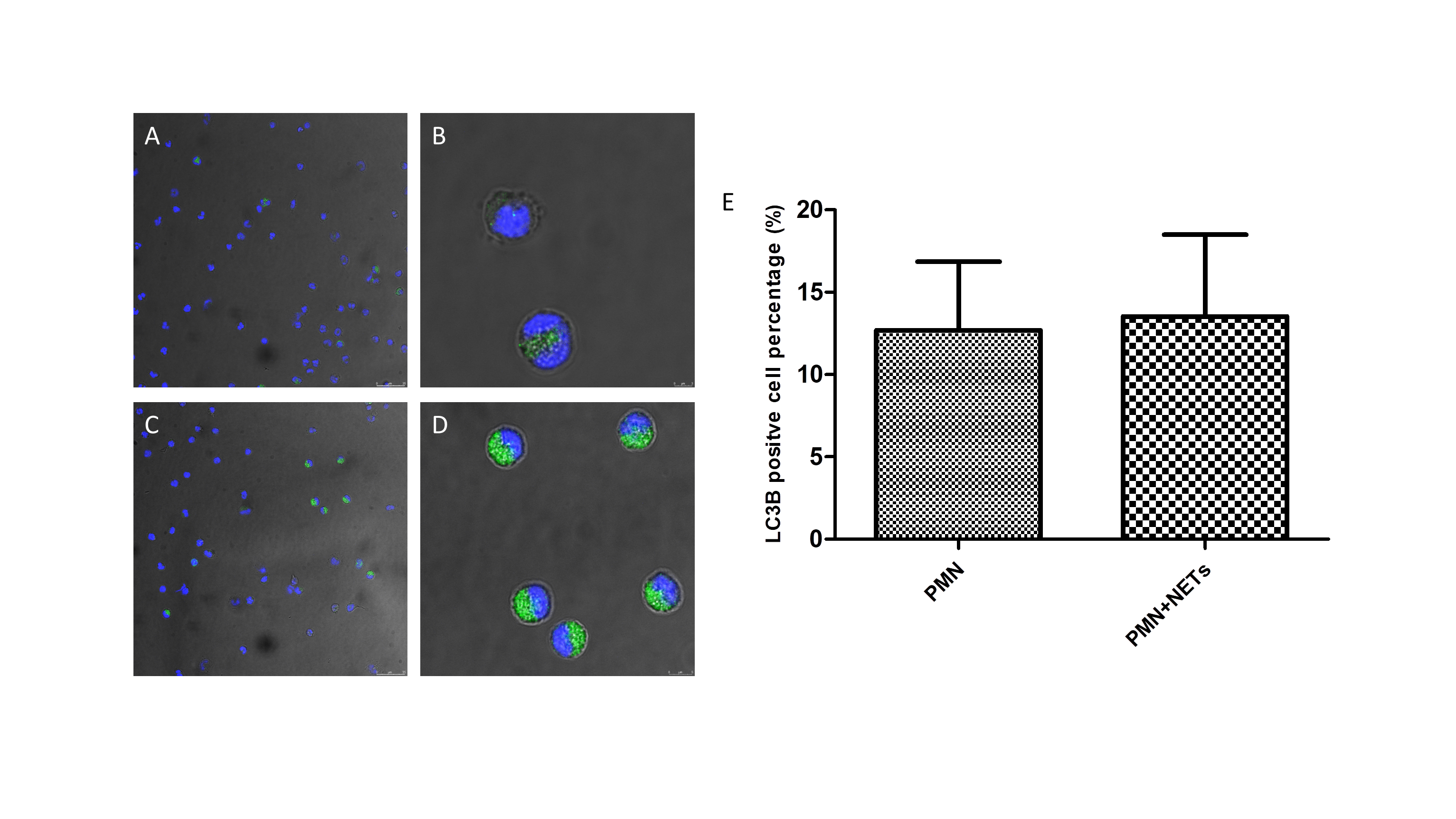

Supplement: Figure S2 — Isolated NETs fails to induce autophagosome formation in bovine PMN. Isolated NETs from B. besnoiti-confronted PMN were isolated as described by Barrientos (59). Bovine PMN were treated with isolated NETs for 1 h, and bovine PMN treated with media was used as control. Samples were fixed and permeabilized with pure cold methanol for LC3B-based immunostaining to determine autophagosome formation by confocal microscopy. (A,B) showing merged images with the staining for LC3B (green), DAPI (blue) in the control group. (C,D) showing merged images with the staining for LC3B (green), DAPI (blue) in the treated group. The right graph shows the percentage of autophagosome-positive cells (E). [file Image_2.TIF]

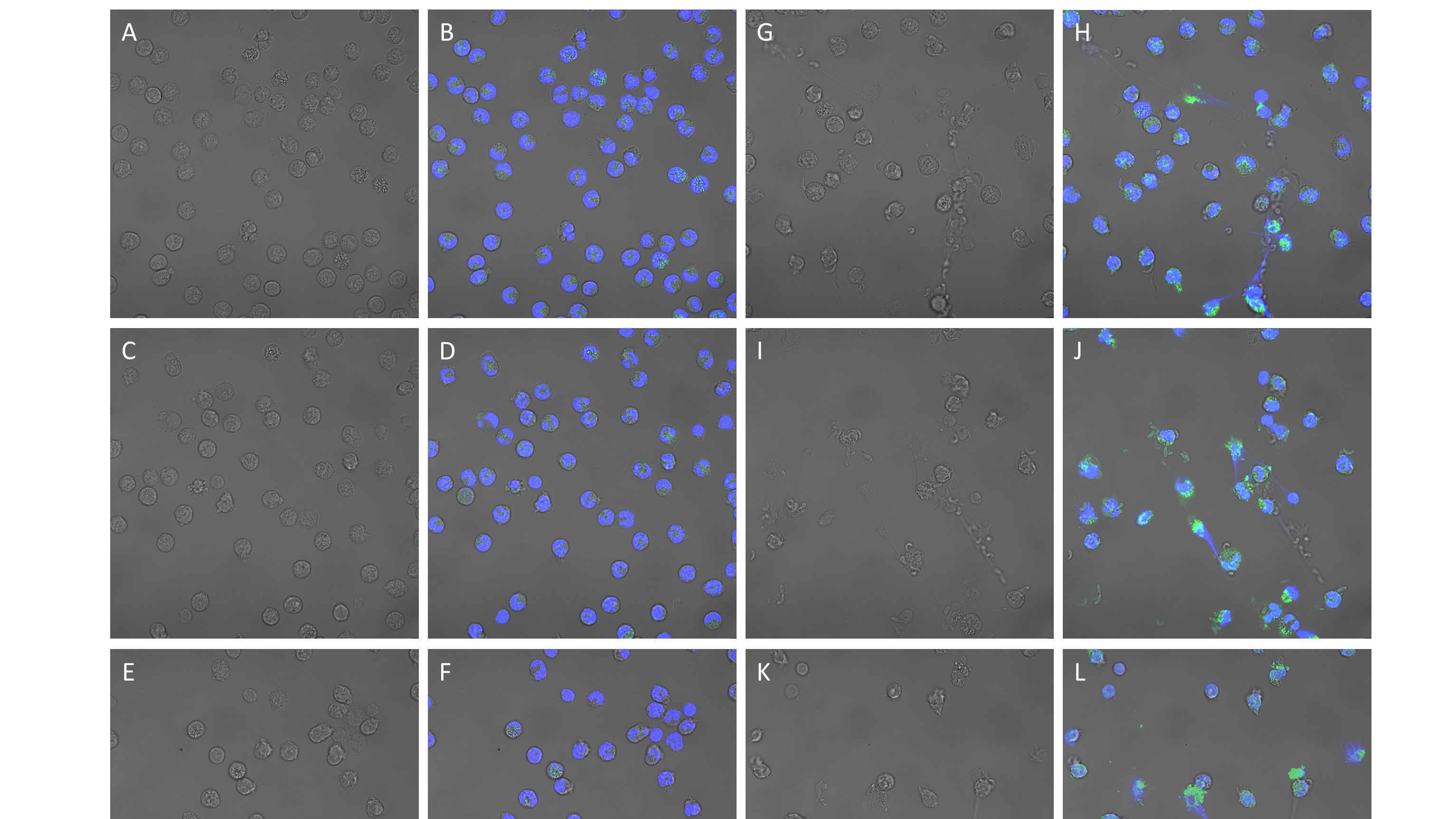

Supplement: Figure S3 — Autophagy and NET formation occurs simultaneously in B. besnoitia-exposed PMN. Bovine PMN were exposed to B. besnoitia tachyzoites for 3 h. Samples were fixed and permeabilized for LC3B-based immunostaining to determine autophagosome formation by confocal microscopy. (A–F) control group: (A,C,E) phase contrast, (B,D,F) merged images; (G–L) PMN+B. besnoiti group: (G,I,K) phase contrast (H,J,L) merged images. Blue: DNA staining with DAPI, green: autophagosomes staining with L3CB antibody. [file Image_3.TIF]

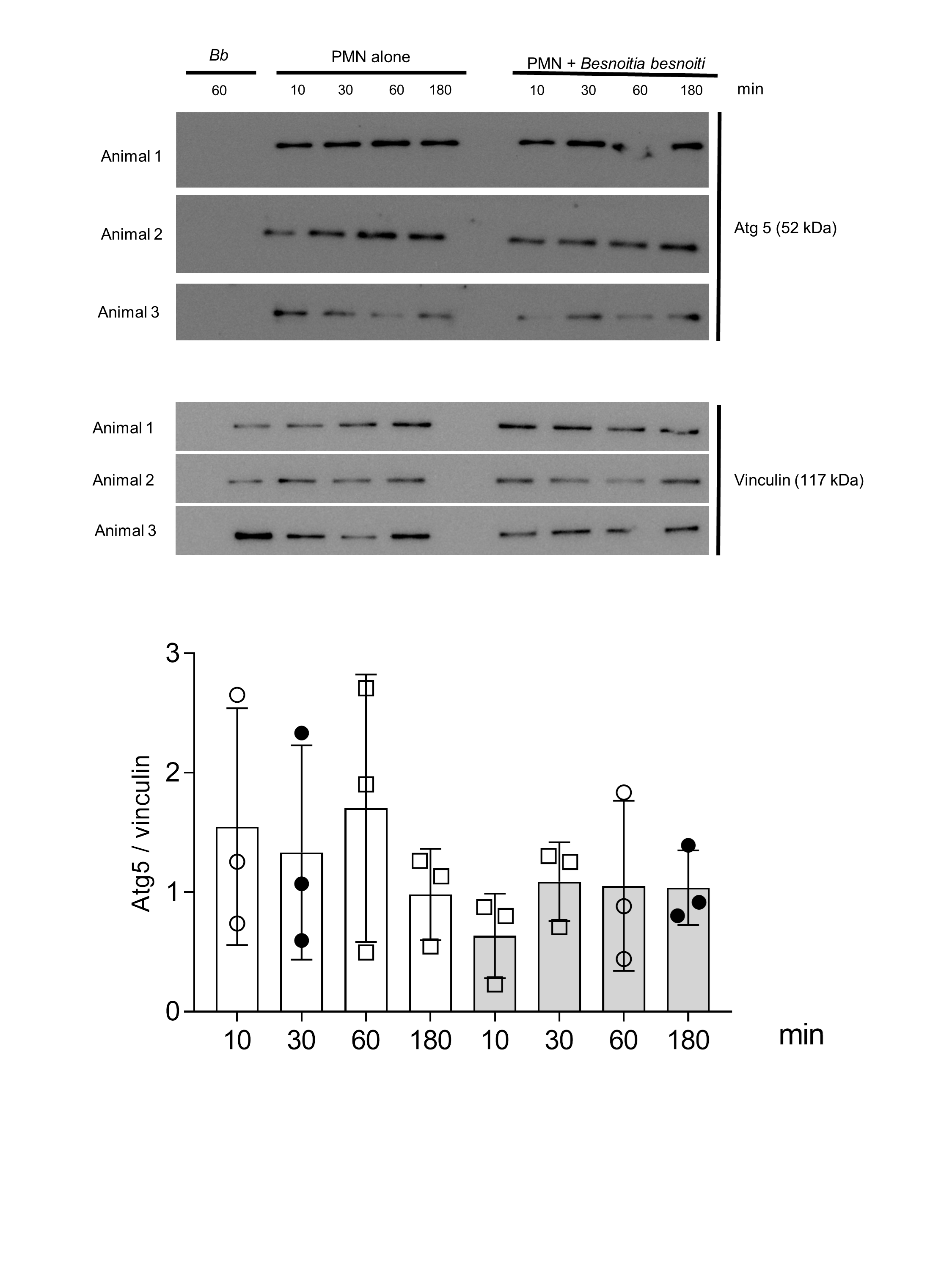

Supplement: Figure S4 — Atg5 protein expression in B. besnoitia-confronted PMN. [file Image_4.TIF]

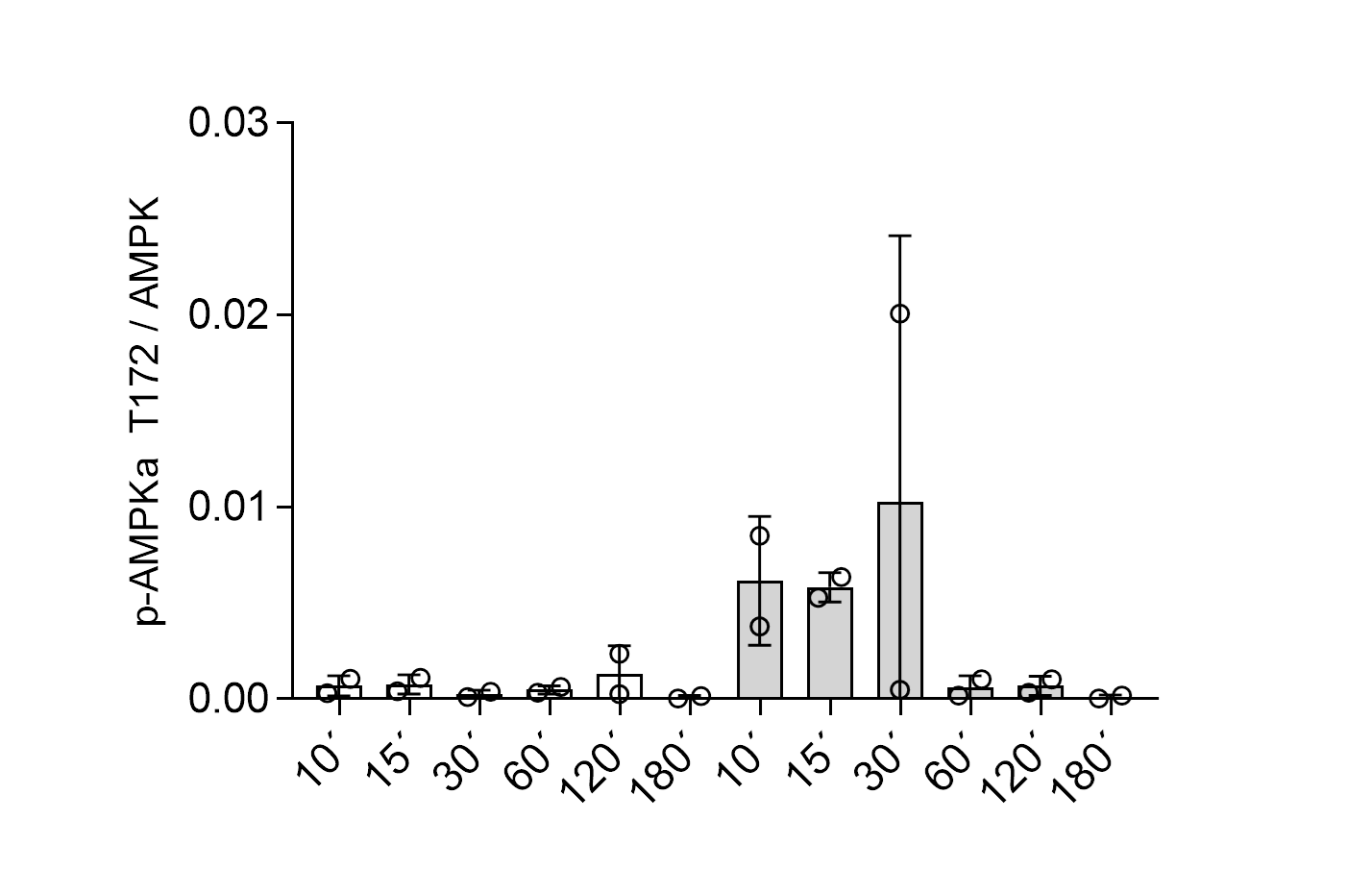

Supplement: Figure S5 — Densitometry quantification of p-AMPKa T127/AMPK. [file Image_5.TIF]

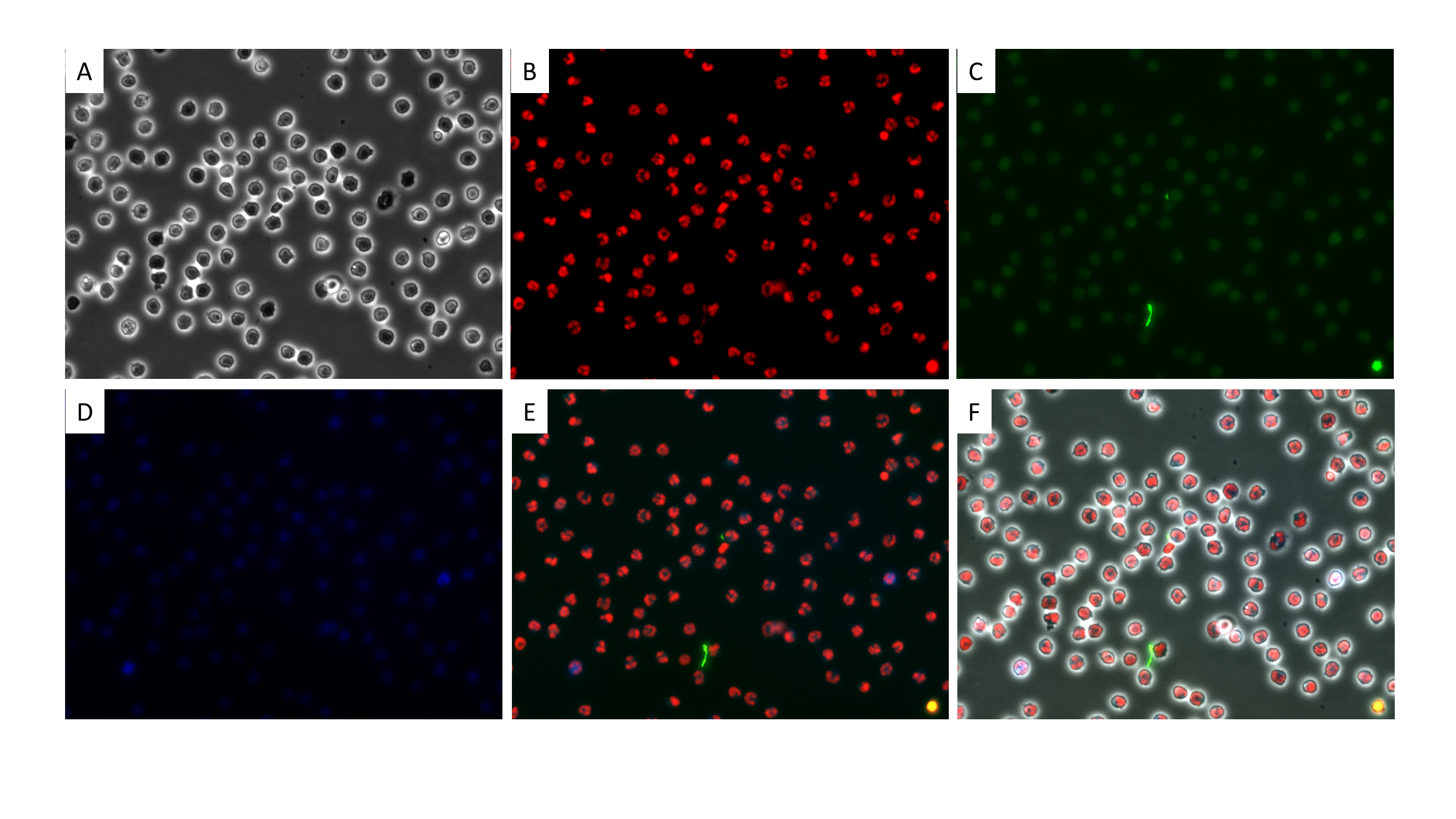

Supplement: Figure S6 — NET formation in control bovine PMN (1/2). Analysis at the same time-point of the experiments performed with B. besnoitia tachyzoites. (A) Phase contrast image; (B) DNA staining: Sytox Orange; (C) histone (H11-4) staining; (D) neutrophil elastase (NE) staining; (E) Merged image of B–D and (F) Merged image of all channels (A–D). [file Image_6.TIF]

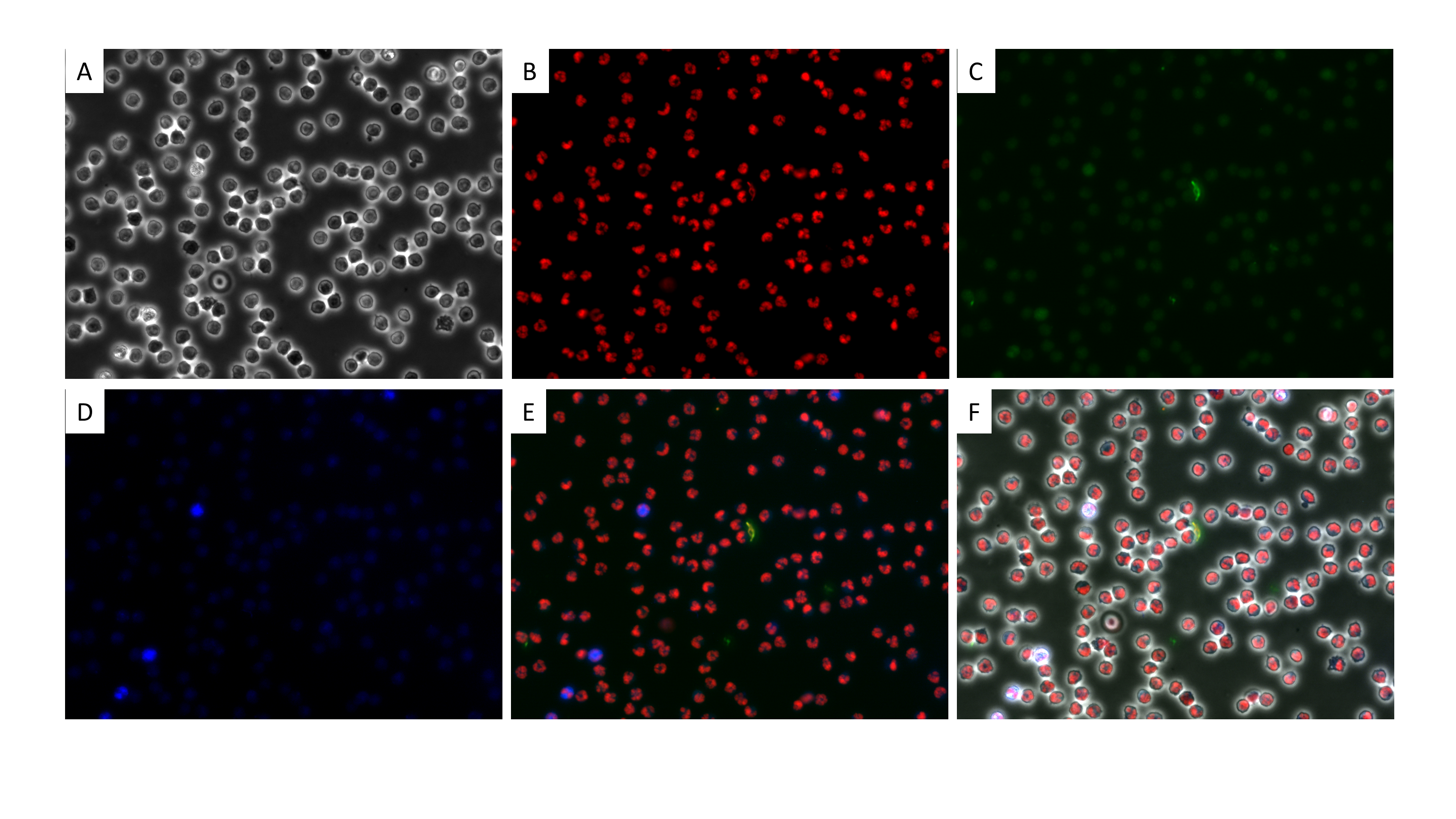

Supplement: Figure S7 — NET formation in control bovine PMN (2/2). Analysis at the same time-point of the experiments performed with B. besnoitia tachyzoites. (A) Phase contrast image; (B) DNA staining: Sytox Orange; (C) histone (H11-4) staining; (D) neutrophil elastase (NE) staining; (E) Merged image of B–D and (F) Merged image of all channels (A–D). [file Image_7.TIF]
